# Inflammation and neutrophil extracellular traps in cerebral cavernous malformation

**DOI:** 10.1007/s00018-022-04224-2

**Published:** 2022-03-25

**Authors:** Anthony C. Y. Yau, Maria Ascencion Globisch, Favour Chinyere Onyeogaziri, Lei L. Conze, Ross Smith, Suvi Jauhiainen, Monica Corada, Fabrizio Orsenigo, Hua Huang, Melanie Herre, Anna-Karin Olsson, Matteo Malinverno, Veronica Sundell, Behnam Rezai Jahromi, Mika Niemelä, Aki Laakso, Cecilia Garlanda, Alberto Mantovani, Maria Grazia Lampugnani, Elisabetta Dejana, Peetra U. Magnusson

**Affiliations:** 1 Department of Immunology, Genetics and Pathology, The Rudbeck Laboratory, Uppsala University, Dag Hammarskjoldsv. 20, 751 85 Uppsala, Sweden; 2 Vascular Biology Unit, The FIRC Institute of Molecular Oncology Foundation, Milan, Italy; 3 Department of Medical Biochemistry and Microbiology, Uppsala University, Uppsala, Sweden; 4 Department of Neurosurgery, University of Helsinki and Helsinki University Hospital, Helsinki, Finland; 5 Department of Biomedical Sciences, Humanitas University, Milan, Italy; 6 IRCCS Humanitas Research Hospital, Milan, Italy; 7 The William Harvey Research Institute, Queen Mary University of London, London, UK; 8 Mario Negri Institute for Pharmacological Research, 20157 Milan, Italy

**Keywords:** Inflammation, Neutrophil extracellular traps, Endothelial cells, Cerebral cavernous malformations

## Abstract

Cerebral Cavernous Malformation (CCM) is a brain vascular disease with various neurological symptoms. In this study, we describe the inflammatory profile in CCM and show for the first time the formation of neutrophil extracellular traps (NETs) in rodents and humans with CCM. Through RNA-seq analysis of cerebellum endothelial cells from wild-type mice and mice with an endothelial cell-specific ablation of the *Ccm3* gene (*Ccm3*^*iECKO*^), we show that endothelial cells from *Ccm3*^*iECKO*^ mice have an increased expression of inflammation-related genes. These genes encode proinflammatory cytokines and chemokines, as well as adhesion molecules, which promote recruitment of inflammatory and immune cells. Similarly, immunoassays showed elevated levels of these cytokines and chemokines in the cerebellum of the *Ccm3*^*iECKO*^ mice. Consistently, both flow cytometry and immunofluorescence analysis showed infiltration of different subsets of leukocytes into the CCM lesions. Neutrophils, which are known to fight against infection through different strategies, including the formation of NETs, represented the leukocyte subset within the most pronounced increase in CCM. Here, we detected elevated levels of NETs in the blood and the deposition of NETs in the cerebral cavernomas of *Ccm3*^*iECKO*^ mice. Degradation of NETs by DNase I treatment improved the vascular barrier. The deposition of NETs in the cavernomas  of patients with CCM confirms the clinical relevance of NETs in CCM.

**Supplementary Information:**

The online version contains supplementary material available at 10.1007/s00018-022-04224-2.

## Introduction

Cerebral cavernous malformation (CCM) is a neurovascular disorder that is characterised by clusters of dilated microvessels mainly in the central nervous system [1]. These malformations can result in various neurological symptoms, including headaches, focal neurological deficits and seizures which are often related to minor bleeding in or around CCM lesions. Currently, diagnosis is commonly made by magnetic resonance imaging and the gold standard of treatment is microsurgical removal for accessible lesions. The results of radiotherapy are contradictory but there is some evidence that patients with multiple symptomatic cavernomas may benefit from therapeutic treatment of propranolol [2, 3].

CCM is a disease of proven genetic origin that can arise sporadically or can be inherited as an autosomal dominant condition with incomplete penetrance [4]. While the sporadic forms of CCM develop only one lesion, the genetic forms of CCM develop multiple vascular lesions that can increase in number and size with age. The difference in lesion burdens between sporadic and familial CCM, as well as genetic analysis of CCM cases [5–7], suggests that CCM pathogenesis may follow the two-hit, biallelic molecular mechanism with somatic and germline mutations. Somatic mutations have been found in a growing number of genes, including *KRIT1* *(CCM1)*, *OSM* *(CCM2)*, *PDCD10 (CCM3)*, *MAP3K3* and *PIK3CA*, and studies of these genes provides further insight into the disease mechanisms [5–11]. Other possible factors contributing to focal vascular malformations in the brain and the spinal cord vasculature include local cellular stresses like disturbed blood flow, oxidation, and inflammation [12–14].

Genetic studies have shown that loss-of-function mutations in *CCM1*, *CCM2*  or (*CCM3* result in CCM. We have previously demonstrated that ablation of the *Ccm* genes in endothelial cells induces endothelial-to-mesenchymal transition (EndMT), which contributes to vascular malformations [15–18], and that the cavernomas develop through clonal expansion of a few *Ccm3*-null endothelial cells [19, 20]. Recently, our group used single-cell RNA sequencing (RNA-seq) to study transcriptional diversity of endothelial cells in vascular cavernomas and showed that it is the endothelial cells of venous and tip cell origin that are responsible for the formation of cavernomas, while endothelial cells of arterial origin are resistant to the *Ccm3* deletion [21].

Over the past 10 years or so, different studies have suggested that inflammation is involved in the pathogenesis of CCM. Recently, a comprehensive transcriptome and gene ontology enrichment analysis of CCM across three species (*C. elegans*, mouse, and human) and two disease genotypes (*Ccm1* and *Ccm3*) [22] identified enriched immune-related gene ontology terms such as neutrophil-mediated immunity and responses to oxidative stress. Transcriptomic analysis of microdissected lesional neuromuscular units of chronic CCM similarly detected differentially expressed genes clustered in inflammation and immune response function [23]. Our recent single-cell RNA-seq study which determined the functional impact of the *Ccm3* deletion in endothelial cells further showed that it is mainly endothelial cells of venous/venous capillary and tip cell origin that had altered immune response profile [21].

Furthermore, the expression of certain human leukocyte antigen types [24] and genetic variations in inflammatory and immune response pathways have been shown to correlate with CCM [25, 26]. Consistent with a role of inflammation in CCM, different leukocyte subsets, including T cells, B cells, and macrophages, have been detected within CCM lesions, and in different murine models of CCM [27–29]. In particular, B-cell depletion has been shown to reduce disease progression in a murine model of CCM [28]. More recently, the use of immunosuppressants or anti-inflammatory agents has been shown to reduce disease levels in murine models of CCM [30–33]. The finding that endothelial-specific deletion, as well as pharmacological suppression of pro-inflammatory innate immunity receptor Toll-like receptor 4 (TLR4) suppress CCM in mice, further supports the involvement of the immune system in CCM pathogenesis [34]. Consistent with this, plasma biomarkers of inflammation have been reported to reflect seizure and recent haemorrhagic activity, and to predict future clinical activity in CCM patients [35, 36].

In this study, we utilize murine models of CCM based on tamoxifen-inducible *Cre* activity, which results in *Ccm3* deletion in endothelial cells in neonatal mice [17, 20, 30]. Here, we combined a number of different approaches to study the inflammatory changes during CCM. These included transcriptomic and proteomic analysis, and immunofluorescence and flow cytometry analysis of the cerebellum from wild-type (control) mice and from mice with endothelial-cell-specific ablation of the *Ccm3* gene (*Ccm3*^*iECKO*^ mice). We thus show that neutrophils are one of the first inflammatory immune cell types recruited to lesions in our murine model of chronic CCM. Neutrophils have the capacity to exert antimicrobial mechanisms by releasing neutrophil extracellular traps (NETs), which are composed of chromatin and bactericidal proteins. This is a well-known phenomenon in different diseases, such as cancers [37], sepsis [38] and also in severe COVID-19 [39], but to date is unknown in CCM while it recently has been described in cerebral arteriovenous malformations [40]. Importantly, we have identified NETs as a potential biomarker of CCM and further showed their potential clinical relevance through demonstration of NETs in patients with CCM.

## Results

### ***RNA-seq analysis reveals a higher expression of inflammation-related genes in brain microvascular endothelial cells of Ccm3***^***iECKO***^*** mice***

**Fig. 1:**
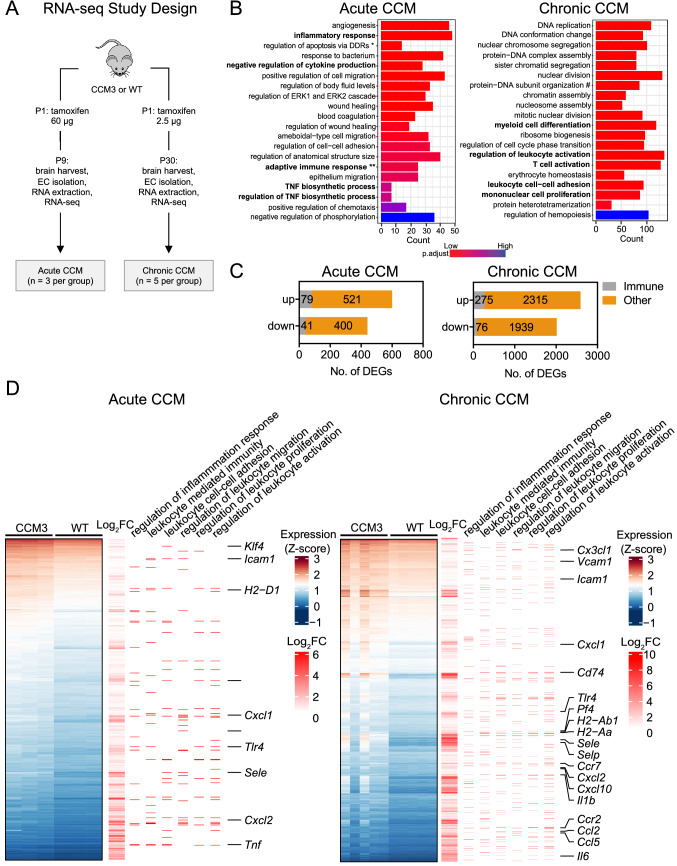
RNA-Seq analysis showed increased expression of inflammation-related genes in brain microvascular endothelial cells from *Ccm3*^*iECKO*^ mice (CCM3) in acute and chronic CCM. **A** Schematic diagram of the experimental design of the transcriptomic study in acute and chronic CCM, comparing CCM3 mice and wild-type (WT) mice (*n* = 3 per group in acute CCM, *n* = 5 in chronic CCM). **B** RNA-Seq gene expression analysis of up-regulated genes showing the top 20 most enriched gene ontology (GO) terms in acute (left panel) and chronic (right panel) CCM. Immune-related GO terms are given in bold. *Regulation of extrinsic apoptotic signaling pathway via death domain receptors, **adaptive immune responses based on somatic recombination of immune receptors built from immunoglobulin superfamily domains, # protein-DNA complex subunit organisation. **C** Bar plots for the numbers of CCM-associated immune genes that were up-regulated or down-regulated (DEGs) in acute (left) and chronic (right) CCM. For definition of immune genes, see Methods. **D** Heatmap showing the expression levels (Z-score of regularized log (rlog)-transformed counts) of significantly up-regulated DEGs (*p*_*adj*_* < *0.05 & |log_2_foldchange|> 0.5; blue: low; red: high) in both acute (upper panel) and chronic (lower panel) models. The first annotation column to the right indicates differential expression in log_2_ fold changes (red: high; white: low). The second to seventh annotation columns indicates the DEGs associated with enriched immune-like GO terms (stated at the top of the figure) from over-representation analysis. Some of the genes labelled after annotation columns were discussed in the text

In this study, we used the murine model of CCM based on tamoxifen-inducible *Cre* activity, for *Ccm3* deletion in endothelial cells in neonatal mice [20, 30]. In the acute, ‘fast progression’ model of CCM (see Methods, Fig. 1A), injection of 60 µg tamoxifen at postnatal day 1 (P1) results in a rapid disease course, with disease onset at around P6, and peak at P8. Most of the animals in the acute model do not survive beyond P10. In the chronic, ‘slow progression’ model of CCM, injection of 2.5–5.0 µg tamoxifen results in a slower disease course, with disease onset at around P7 to P8. Most of these mice reached adulthood, and only a small proportion of the cells (< 15%) show *Ccm3* deletion [20].

To better understand the roles of inflammation in the development of CCM, we performed RNA-seq to study the transcriptome of endothelial cells isolated from the cerebellum from *Ccm3*^*iECKO*^ mice with acute and chronic CCM, compared to the wild-type controls (Fig. 1A). The over-representation analysis of up-regulated differentially expressed genes (DEGs, *p*_*adj*_* < *0.05 & |log_2_foldchange|> 0.5) in CCM compared to wild-type control identified many enriched Gene Ontology (GO) terms that are related to inflammation and immune response. Five of them were among the top 20 on the list of all enriched GO terms when ranked by the adjusted *p* value (Fig. 1B, marked in bold), such as regulation of leukocyte migration, adhesion and proliferation, and cytokine production. Gene Set Enrichment Analysis (GSEA) also identified immune-related gene sets enriched in both acute and chronic CCM, e.g. ‘inflammatory response’ from ‘hallmark’ gene sets (Supplemental Fig. 1).

Using the results from functional analysis as a reference, we extracted a list of immune-related gene sets from the MSigDb collection (see Methods). The DEGs that are associated with these immune-related gene sets were defined as CCM-associated immune genes. In total, we identified 120 (11.5%, out of 1041 DEGs) CCM-associated immune genes in the acute CCM model and 351 (7.6%, out of 4605 DEGs) (7.6%) in the chronic CCM model (Fig. 1C). The over-represented immune-related GO terms among up-regulated DEGs in acute and chronic CCM are shown in Fig. 1D. Some of these genes are known for their encoded functions as chemoattractants for leukocytes, including neutrophils, T cells and monocytes. These genes were *Cxcl1*, *Ccl2*, *Ccr2*, *Ccl5*, *Cxcl2* and *Cx3cl1* (Fig. 1D, Table 1). Other genes encode proteins that mediate the interaction of endothelial cells with leukocytes and platelets, such as *Icam1*, *Vcam1*, *Selp* and *Sele* (Fig. 1D, Table 1). Some of the genes encode proinflammatory cytokines, such as *Il1b*, *Il6* and *Tnf*, and major histocompatibility complex (MHC) class II molecules for antigen presentation, such as *H2-Ab1* (Fig. 1D, Table 1).

**Table 1:** Immune-related differentially expressed genes identified through RNA-Seq of endothelial cells isolated from *Ccm3*^*iECKO*^ mice during acute and chronic CCM, along with some of their functions

Gene	Function
*Il-1b*	Proinflammatory cytokine, promotes activation and acute phase response
*Tnf*	Proinflammatory cytokine, promotes activation and acute phase response
*H2-Ab1*	MHC class II, antigen presentation
*Cxcl1/KC/GRO*	Chemoattractant for neutrophils
*Cxcl2/MIP2a*	Chemoattractant for neutrophils. Expressed at inflammation sites and might suppress haematopoietic progenitor cell proliferation
*Cx3cl1*	Chemoattracts T cells and monocytes; promotes adhesion of leukocytes to activated endothelial cells
*Ccl2/MCP-1*	Shows chemotactic activity for monocytes and basophils
*Ccl3/Mip1a*	Involved in recruitment and activation of polymorphonuclear leukocytes
*Vcam1*	Mediates leukocyte-endothelial cell adhesion and signal transduction
*Icam1*	Upon activation, leukocytes bind to endothelial cells via ICAM-1/LFA-1 and transmigrate into tissues
*P-selectin (Selp)*	Mediates interaction of activated endothelial cells or platelets with leukocytes
*E-selectin (Sele)*	Mediates adhesion of neutrophils in activated endothelium through interaction with SELPLG/PSGL1

**Fig. 2:**
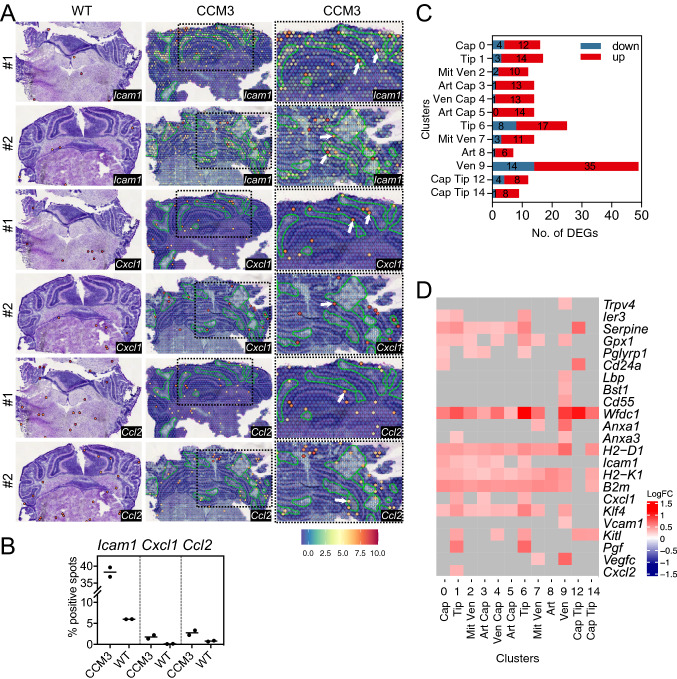
Spatial transcriptomics datashowed an increased expression of genes that encode proteins with chemoattractant and adhesion functions. By scRNA sequencing analysis venous/venous capillary endothelial cells showed the highest number of upregulated and downregulated CCM-associated immune genes. **A** Gene expression levels of *Icam1*, *Cxcl1* and *Ccl2* measured in Visium spatial transcriptomics data of *Ccm3*^*iECKO*^ mice (CCM3) in acute CCM. All sequenced dots are shown, colour coded for expression: blue, low; red, high. Negative spot outlines are shown without fill colour for visualisation of underlying haematoxylin and eosin staining (dark, light violet). Both complete sections and magnified boxed areas are shown. Green lines, outline of CCM lesions. Arrows, examples of spots expressing the indicated genes that colocalised with, or were in close proximity to, the periphery of the cavernomas. **B** Plots showing proportions (%) of spots in the cerebellum expressing *Icam1*, *Cxcl1* and *Ccl2* measured in Visium spatial transcriptomics data, as shown in **A** (*n* = 2 per group). **C** Numbers of CCM-associated immune genes (Fig. 1C, see method) by scRNA sequencing analysis (scRNA-seq) that were up-(red) or down-(blue) regulated (*p*_*adj*_* < *0.05) in each endothelial cell subtype of *Ccm3*^*iECKO*^ mice in acute CCM [21]. **D** Heatmap showing log fold expression changes of immune response associated genes identified from bulk RNA-seq (Fig. 1D, gene annotation column) in each cell type of scRNA-seq data [21]. Only immune response genes that differed in gene expression between *Ccm3*^*iECKO*^ and wild-type (WT) control mice in at least one endothelial cell subtype are shown here. **C–D** The identify of each endothelial cell subtype cluster (**C**) is as follows: *Cap* capillary (C0), *Tip* tip cells (C1, C6), *Mit Ven* mitotic/venous capillary (C2, C7), *Art Cap* arterial capillary (C3, C5), *Ven Cap* venous capillary (C4), *Art* arterial (C8), *Ven* venous/venous capillary (C9), *Cap Tip* capillary/tip cells (C12, C14)

Similar observations can be found in our previously published spatial transcriptomics data [21] (acute CCM model; P8 mouse brain cryosections of *Ccm3*^*iECKO*^ mice and wild-type control) where the proportion of spots in the cerebellum that express *Cxcl1*, *Ccl2*, and *Icam1* genes was much higher in the *Ccm3*^*iECKO*^ mice than that of the wild-type mice (Fig. 2A). Interestingly, many spots in our spatial transcriptomic data, which highly express *Cxcl1*, *Ccl2*, or *Icam1*, were co-localised with, or in close proximity to, the periphery of the cavernomas (Fig. 2B, examples of positive spots indicated by white arrowheads, and lesions outlined by greenish-blue lines). Since the chemokine CXCL1 and the cytokine CCL2 have chemoattractant roles and ICAM1 induces leukocyte interactions, their high expression in the periphery of cavernomas suggested a role in leukocyte recruitment and leukocyte endothelial transmigration for the endothelial cells lining the cavernomas.

To further investigate the contributions of the different endothelial cell subtypes to the observed inflammation, we first examined the CCM-associated immune genes identified from the bulk RNA-seq (Fig. 1C) in our previously published scRNA-seq dataset [21], where the subtypes of brain endothelial cells under both normal (wild-type) and CCM (*Ccm3*^*iECKO*^) conditions in the acute model of CCM were comprehensively characterized. Figure 2C shows the number of differentially regulated CCM-associated immune genes between *Ccm3*^*iECKO*^ and wild-type cells in each endothelial cell subtype (*p*_*adj*_* < *0.05). Venous/venous capillary endothelial cells (cluster 9) seemed to be the most inflammatory/immune responsive to the deletion of *Ccm3* among all endothelial subtypes with 35 upregulated and 14 downregulated CCM-associated immune genes. Arterial endothelial cells (cluster 8), on the contrary, responded the least to the *Ccm3* deletion with only a few inflammatory genes affected (6 upregulated; 1 downregulated). Other endothelial cells subtypes showed inflammatory/immune responsive intermediate between venous/venous capillary and arterial endothelial cells.

Next, we checked the regulation of the immune response associated genes (Fig. 1D) in the scRNA-seq dataset [21]. Similar to CCM-associated immune genes, venous/venous capillary endothelial cells (cluster 9) have the most immune response associated genes changed between *Ccm3*^*iECKO*^ and wild-type conditions, while arterial endothelial cells (cluster 8) have the least genes changed. It indicates that venous/venous capillary endothelial cells were more inflammatory/immune responsive than arterial endothelial cells (cluster 8 in Fig. 2D and Supplemental Fig. 2). Furthermore, we identified several genes with known immune functions that were upregulated only in the venous/venous capillary endothelial cells (cluster 9 in Fig. 2D), but not in other endothelial cell subtypes, including *Lbp* (known for encoding a protein involved in acute phase response in innate immunity), *Cd55* (encodes a glycoprotein involved in the regulation of the complement cascade), and *Vcam1* (encodes a membrane protein that mediates leukocyte-endothelial cell adhesion and signal transduction). Two other immune genes, *Anxa1* and *Anxa3*, both encode proteins that inhibit phospholipase A2 and has anti-inflammatory activity, are upregulated in only venous/venous capillary (cluster 9) and mitotic/venous capillary endothelial cells (cluster 7) for *Anxa1* or tip cells (cluster 1) for *Anxa3*, but not in other endothelial cell subtypes.

### ***Increased levels of cytokines and chemokines are mainly associated with neutrophils and monocytes in the cerebellum of Ccm3***^***iECKO***^*** mice***

As the transcriptomic analysis showed an up-regulation of inflammation-related genes in acute and chronic CCM, including those encoding proinflammatory cytokines and chemokines, we next aimed to determine the protein levels of some of these inflammatory cytokines and chemokines in the cerebellum of *Ccm3*^*iECKO*^ mice. Previous studies in CCM patients have demonstrated clinical associations of plasma biomarkers of inflammation, including inflammatory cytokines [35, 36]. Nevertheless, the levels of these inflammatory biomarkers have not been investigated locally in the brain in CCM.

**Fig. 3:**
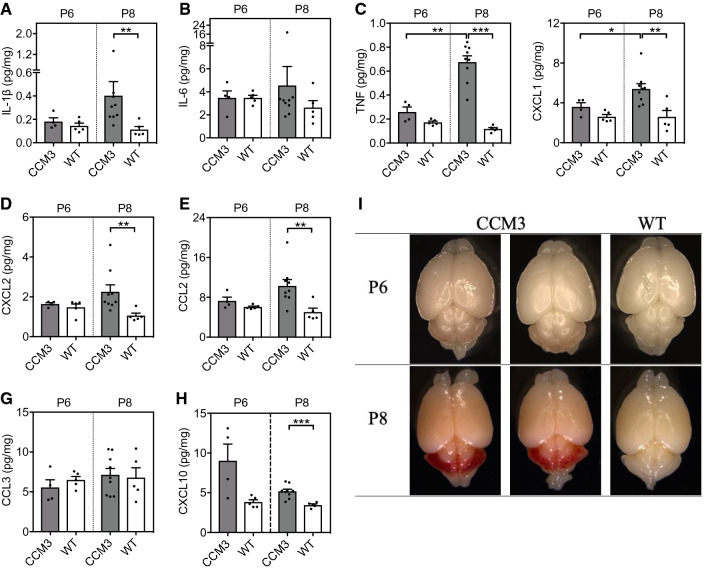
Proinflammatory cytokines and chemokines mainly associated with recruitment of neutrophils and monocytes were elevated in the cerebellum of *Ccm3*^*iECKO*^ mice (CCM3) compared with wild-type controls (WT) during acute CCM. The following cytokines and chemokines are shown: IL-1β (**A**), IL-6 (**B**), TNF (**C**), CXCL1/KC/GRO (**D**), CXCL2/MIP-2 (**E**), CCL2/MCP-1 (**F**), CCL3/MIP-1α (**G**) and CXCL10/IP-10 (**H**), at P6 (*n* = 4–5 per group) and P8 (*n* = 5–9 per group). **I** Representative images of the whole brain at P6 and P8. *P < 0.05; **P < 0.01; ***P < 0.001, for comparisons between groups (Mann–Whitney *U* tests)

Using multiplexed, high-sensitivity, quantitative cytokine assays, we determined the levels of 19 different cytokines and chemokines in the cerebellum tissues of wild-type control and *Ccm3*^*iECKO*^ mice, for both acute and chronic CCM. In acute CCM, the measurements were performed at disease onset (P6) and at disease peak (P8) (Fig. 3A–I). At P6, there were no differences in the levels of any of the cytokines and chemokines (Fig. 3A–H, Supplemental Fig. 3A–J). At P8, however, there were increased levels in six of the tested cytokines and chemokines in the *Ccm3*^*iECKO*^ mice compared to the wild-type controls. These elevated cytokines and chemokines were: IL-1β, TNF, CXCL1/KC/GRO, CXCL2/MIP-2, CCL2/MCP-1 and CXCL10/IP-10 (Fig. 3A–H, Supplemental Fig. 3A–J).

**Fig. 4:**
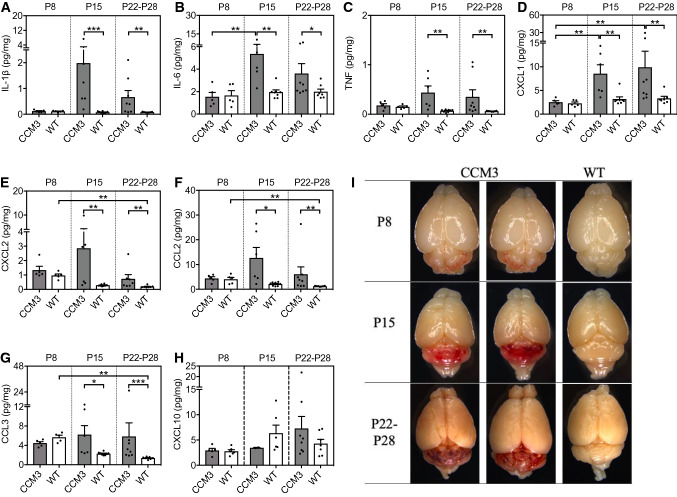
Proinflammatory cytokines and chemokines mainly associated with recruitment of neutrophils and monocytes were elevated in the cerebellum of *Ccm3*^*iECKO*^ mice (CCM3) compared with wild-type controls (WT) during chronic CCM. The following cytokines and chemokines are shown: IL-1β (**A**), IL-6 (**B**), TNF (**C**), CXCL1/KC/GRO (**D**), CXCL2/MIP-2 (**E**), CCL2/MCP-1 (**F**), CCL3/MIP-1α (**G**) and CXCL10/IP-10 (**H**), at P8 (*n* = 5–6 per group), P15 (*n* = 6–8 per group) and P22-P28 (*n* = 7–8 per group). **I** Representative images of the whole brain at P8, P15 and P22-28. *P < 0.05; **P < 0.01; ***P < 0.001 for comparisons between groups. Mann–Whitney *U* tests were used to determine statistically significant differences in the levels of cytokines and chemokines between *Ccm3*^*iECKO*^ and WT mice. Kruskal–Wallis test ANOVA with Dunn’s multiple comparisons test were used to determine statistically significant differences in the levels of cytokines and chemokines between different time points in *Ccm3*^*iECKO*^ mice

In chronic CCM, the measurements were performed at disease onset (P8), at the developing phase of the disease (P15) and near the disease peak (P22-P28) [35, 36]. At P8, there were no differences in the levels of any of the cytokines and chemokines (Fig. 4A–I, Supplemental Fig. 4A–J). At P15, and P22-P28, however, there were significant increases in seven of the cytokines and chemokines in the *Ccm3*^*iECKO*^ mice compared with the wild-type controls. These were: IL-1β, IL-6, TNF, CXCL1/KC/GRO, CXCL2/MIP-2, CCL2/MCP-1 and CCL3/MIP-1α (Fig. 3A–H, Supplemental Fig. 4A–J).

For both acute and chronic CCM, among the 19 cytokines and chemokines that were evaluated using the multiplexed, high-sensitivity, quantitative cytokine assays, no differences were seen for 10 of them for either acute or chronic CCM: IL-2, IL-4, IL-5, IL-10, IL-12, IL-15, IL-17A, IL-27, IL-33 and IFN-γ (Supplemental Figs. 3, 4); the levels of IL-9 were below the detection limit of this system. Five of the 19 tested cytokines and chemokines showed increased levels in both acute and chronic CCM: IL-1β, TNF, CXCL1/KC/GRO, CXCL2/MIP-2 and CCL2/MCP-1; these were consistent with the RNA-seq data. Three of the cytokines and chemokines showed increased levels in either acute or chronic CCM: IL-6, CCL3/MIP-1α and CXCL10/IP-10 (Figs. 3, 4). It is worth noting that most of these elevated cytokines and chemokines were produced by monocytes or neutrophils, or have functions related to the chemotactic activities of monocytes or neutrophils (Table 1).

Next, we investigated the correlation between disease severity in chronic CCM, as determined by lesion size, and the levels of the eight up-regulated cytokines and chemokines: IL-1β, IL-6, TNF, CXCL1/KC/GRO, CXCL2/MIP-2, CCL2/MCP-1, CCL3/MIP-1α and CXCL10/IP-10. Here, we observed strong correlation between disease severity of CCM and the levels of these cytokines and chemokines (Supplemental Fig. 5A–H).

### ***Immune cell recruitment in the cerebellum of Ccm3***^***iECKO***^*** mice***

As both the transcriptomic and proteomic studies of the cerebellum from *Ccm3*^*iECKO*^ mice indicated an important role for the recruitment of inflammatory cells in CCM development, we investigated this further in this mouse model of CCM. To determine the immunological changes for the different phases of CCM, we investigated further using the chronic model of CCM that has a slower disease progression.

**Fig. 5:**
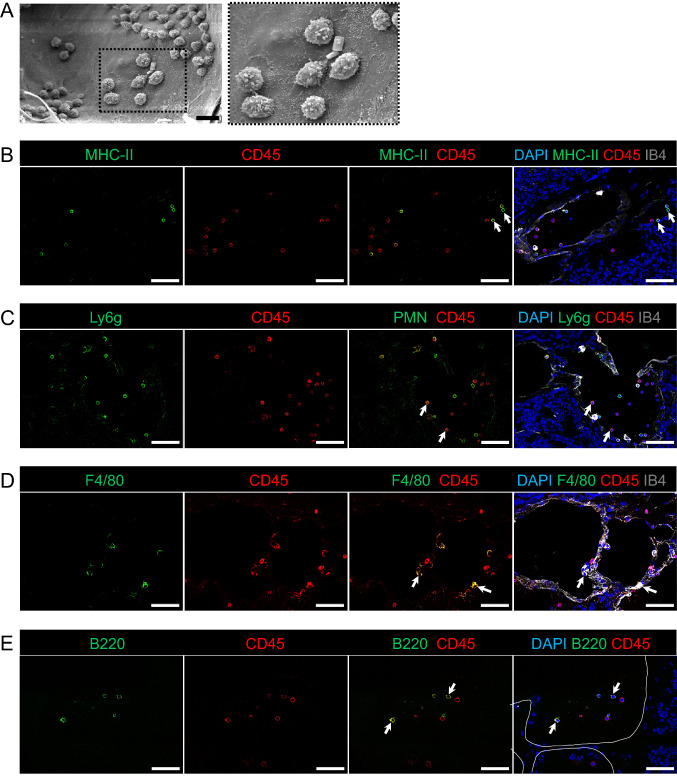
Representative scanning electron microscopy and immunofluorescence showed recruitment of different immune cell subsets at CCM lesions in the cerebellum of the *Ccm3*^*iECKO*^ mice at P28 for chronic CCM. **A** Leukocytes are attached to the endothelial cells of CCM lesions in chronic CCM. **B-E** Cerebellum in *Ccm3*^*iECKO*^ mice at P28 in chronic CCM. DAPI staining (blue), together with: (**B**) MHC class II (green), CD45 (red) and isolectin-B4 (white); (**C**) Ly6g^+^ (green), CD45 (red) and isolectin-B4 (white); (**D**) F4/80 (green), CD45 (red) and isolectin-B4 (white); and (**E**) CD45R/B220 (red) and CD45 (white). White line in (**E**) shows the location of the lesions in the cerebellum; white arrows, examples of the immune cell subsets that are positive for both DAPI and CD45. Scale bars: 10 μm (**A**); 50 μm (**B–E**)

Using scanning electron microscopy of the cerebellum from the *Ccm3*^*iECKO*^ mice, we observed different leukocytes attached to the endothelial cells of the CCM lesions in chronic CCM at P28 (Fig. 5A). Immunofluorescence further confirmed the accumulation of inflammatory leukocytes (positive for both DAPI and CD45) at the lesions in the *Ccm3*^*iECKO*^ mice at P28 for chronic CCM (Fig. 5B–E), similar to previous reports in other models of CCM [29]. Such DAPI^+^ CD45^+^ inflammatory leukocytes were not detected in the wild-type mouse brains (Supplemental Fig. 6). Some of the DAPI^+^ CD45^+^ leukocytes detected in CCM lesions were positive for MHC class II (Fig. 5B). Furthermore, polymorphonuclear neutrophils, F4/80^+^ macrophages, and B220^+^ B cells were also seen in the CCM lesions (Fig. 5C–E, respectively), but not in the surrounding normal brain tissue.

### ***Quantitative changes in inflammatory cells in the cerebellum of Ccm3***^***iECKO***^*** mice***

**Fig. 6:**
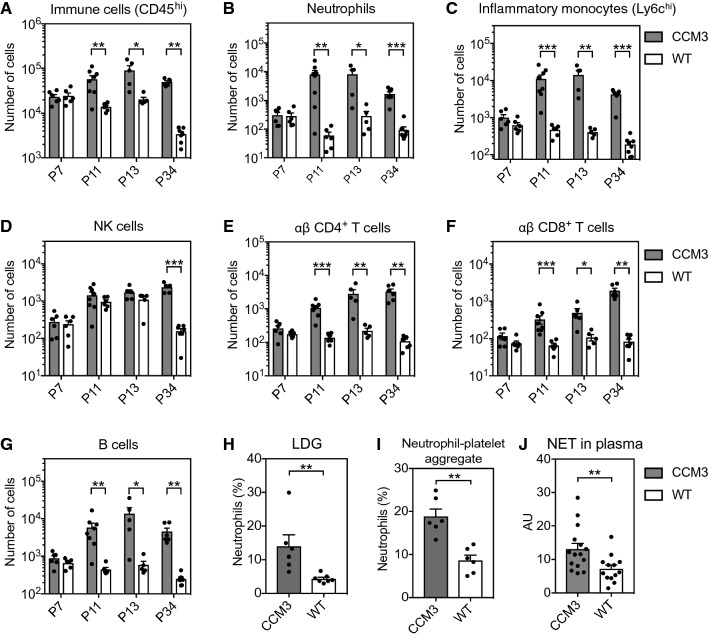
Flow cytometry analysis showed that different leukocyte subsets were elevated in cell numbers in the cerebellum of *Ccm3*^*iECKO*^ mice (CCM3) compared with wild-type controls (WT) during chronic CCM. Activated neutrophils, neutrophil–platelet aggregates and NET levels were elevated in circulation in *Ccm3*^*iECKO*^ mice (CCM3) compared with wild-type controls (WT) during chronic CCM. The following are shown: immune cells (CD45^hi^) (**A**), neutrophils (CD45^hi^CD11b^+^Ly6g^+^) (**B**), inflammatory monocytes (CD45^hi^CD11b^+^Ly6g^+^Ly6c^hi^) (**C**), natural killer (NK) cells (CD45^hi^CD11b^−^CD19^−^NK1.1^+^CD3^−^) (**D**), CD4 T cells (CD45^hi^CD11b^−^CD19^−^NK1.1^−^γδΔTCR^−^CD3^+^CD4^+^) (**E**), CD8 T cells (CD45^hi^CD11b^−^CD19^−^NK1.1^−^ γδTCR^−^CD3^+^CD4^−^) (**F**) and B cells (CD45^hi^CD11b^−^CD19^+^NK1.1^−^) (**G**), at P7 (*n* = 6 per group), P11 (*n* = 6–8 per group), P13 (*n* = 5 per group) and P34 (*n* = 7–8 per group). Low-density granulocytes (LDG) (**H**), and neutrophil–platelet aggregates **(I)** as a proportion (%) of circulating neutrophils (CD45^+^CD11b^+^Ly6c^lo^Ly6g^+^) in wild-type controls and *Ccm3*^*iECKO*^ mice (*n* = 6–7 per group) during chronic CCM at P13. **J** Neutrophil extracellular traps (NETs) in wild-type controls and *Ccm3*^*iECKO*^ mice (*n* = 13–15 per group) during chronic CCM, at P15, expressed as arbitrary units (AU). *P < 0.05; **P < 0.01; ***P < 0.001 for comparisons between groups (Mann–Whitney *U* tests)

To better understand the dynamic changes in the numbers of immune cells during CCM development, flow cytometry analysis was performed for single cell suspensions of freshly collected cerebellum from the wild-type controls and the *Ccm3*^*iECKO*^ mice (Supplemental Fig. 7). Different phases of CCM development were examined, from the onset of disease (P7), through the developing phases (P11, P13), to the fully established disease (P34) (Fig. 6, Supplemental Fig. 8) [20]. At P7, there were no differences in the numbers of immune cells between the *Ccm3*^*iECKO*^ mice and the wild-type controls. At P11, there was a dramatic increase in the numbers of different immune cell populations in the cerebellum of the *Ccm3*^*iECKO*^ mice, including neutrophils, macrophages/monocytes, B cells and T cells (Fig. 6, Supplemental Fig. 8). These increased numbers for the different immune cells continued to P34 in chronic CCM. The ratios were also calculated for the numbers of immune cells for *Ccm3*^*iECKO*^ mice to the wild-type controls for each immune cell type (Supplemental Fig. 9). Among these, neutrophils and inflammatory monocytes showed the highest ratios at the earlier times of P11 and P13, respectively, which were then reduced at P34. On the contrary, the ratios of immune cells associated with adaptive immunity, such as the CD4^+^ and CD8^+^ T cells, increased with disease development, and were highest at P34 (Supplemental Fig. 9).

### *Increased frequencies of activated neutrophils, NETs, and neutrophil–platelet aggregates, in chronic CCM*

As both transcriptomic and proteomic analyses showed increased chemoattractant activities for neutrophils in CCM (Figs. 1–4), and both immunofluorescence and flow cytometry analyses showed recruitment of large numbers of neutrophils to the cavernoma in chronic CCM (Figs. 5, 6), the properties and roles of neutrophils in CCM development were further characterised. In general, neutrophils are among the first cells that are recruited to inflammatory sites. Neutrophils have been described as having multiple effector mechanisms, including phagocytosis and the production of reactive oxygen species, proteases and NETs.

The pathological features of the neutrophils in CCM were thus initially characterised here. Flow cytometry analysis was carried out for blood from the wild-type controls and the *Ccm3*^*iECKO*^ mice (Supplemental Fig. 10). With gating for neutrophils (i.e., CD45^+^CD11b^+^Ly6c^lo^Ly6g^+^), there was a population of high-scatter cells that were more frequent among the neutrophils from the *Ccm3*^*iECKO*^ mice compared to the wild-type control (Fig. 6H). These enlarged cells have been described as activated neutrophils [41] or low-density granulocytes [42], and they have been shown to generate increased levels of NETs under autoimmune conditions [43]. Furthermore, there were higher frequencies of neutrophils that formed aggregates with platelets (i.e., CD41^+^ neutrophils) in the *Ccm3*^*iECKO*^ mice compared to the wild-type control (Fig. 6I). This is of particular interest, as neutrophil–platelet interplay has been described previously as the key neutrophil response in different inflammatory processes, and as platelets have been shown to induce NET formation [44, 45]. Consistent with this, citrullinated histone H3 (citH3)-DNA ELISA analysis of plasma samples for NET formation in *Ccm3*^*iECKO*^ mice and wild-type mice showed that the *Ccm3*^*iECKO*^ mice had higher plasma levels of NETs compared to the wild-type mice (Fig. 6J).

**Fig. 7:**
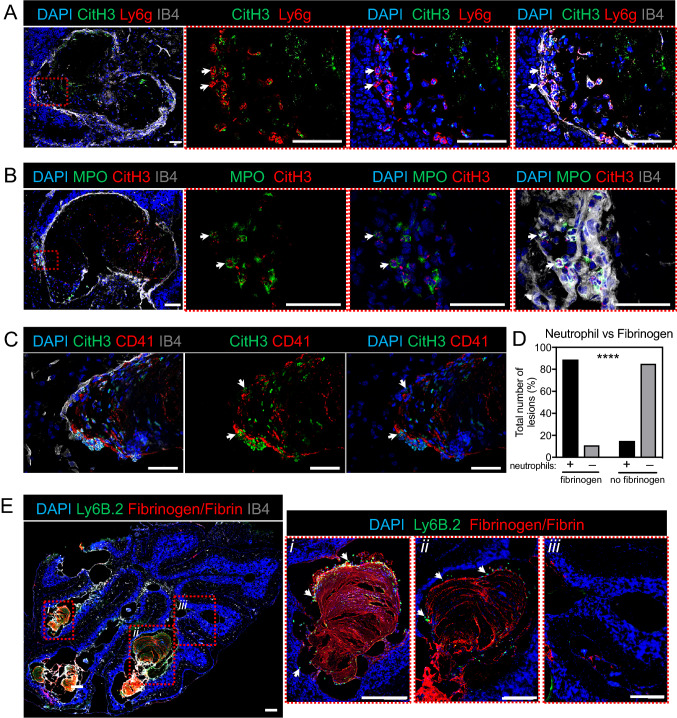
Characterisation of neutrophils and neutrophil extracellular traps (NETs) in the cerebellum at P15 during chronic CCM. **A–D** Representative immunofluorescence for deposition of NETs in *Ccm3*^*iECKO*^ mice during chronic CCM, at P15. **A** Citrullinated histone H3 (green), Ly6g (red), DAPI (blue) and isolectin B4 (grey). **B** MPO (green), citrullinated histone H3 (red), DAPI (blue) and isolectin B4 (grey). **C** Citrullinated histone H3 (green), CD41 (red), DAPI (blue) and isolectin B4 (grey). **D** Proportions (%) of lesions with (left) or without (right) fibrinogen/fibrin signals that attract (black bar) or do not attract (grey bar) neutrophils. Total number of lesions analysed, 79 (in 4 mice). ****P < 0.0001, for cavernoma without and with fibrinogen/fibrin signals (Fisher’s exact tests). **E** Anti-Ly-6B.2 (green), fibrinogen/fibrin (red), DAPI (blue) and isolectin B4 (grey). White arrows: NETs (**A**–**C**); neutrophils (**E**). Scale bars: 50 μm (**A**–**C**); 200 μm (**E**)

The higher frequency of activated neutrophils and neutrophil–platelet aggregates might thus explain the increased levels of NETs detected in blood in *Ccm3*^*iECKO*^ mice (Fig. 6H–J). We next investigated the presence of NETs in the brain cavernoma of these *Ccm3*^*iECKO*^ mice. Immunofluorescence analysis using antibodies against different NET markers showed that there were NETs in the cavernoma in the cerebellum of these *Ccm3*^*iECKO*^ mice (Fig. 7A, B), but not for the wild-type controls (Supplemental Fig. 11), as seen for Ly6g and MPO (neutrophil marker) and citrulline histone H3. Using co-staining with the platelet marker CD41, colocalisation of citrullinated histone H3 positive signals were seen with platelets in the cavernoma in the cerebellum of the *Ccm3*^*iECKO*^ mice, mainly close to the endothelium (Fig. 7C). Neutrophil–platelet interactions at the activated endothelium can result in occlusion of the microvessels during immunothrombosis, and to thromboinflammation [44, 46], which represents a possible pathological consequence of NET formation in CCM. This is partly supported by our observations that neutrophils are more often found in cavernoma with substantial coagulation (as determined by immunofluorescence staining for fibrinogen/fibrin), compared to cavernoma with little or no coagulation (Fig. 7D, 89% vs 15%, respectively). In Fig. 7E, boxes *i* and *ii* show detection of neutrophils (indicated by staining of Ly-6B.2) in cavernomas with substantial coagulation and neutrophils interacting with fibrinogen (arrows), whereas box *iii* shows the absence of neutrophils in cavernomas with no  fibrinogen/fibrin.

### *Coagulation precedes NET formation in chronic CCM*

**Fig. 8:**
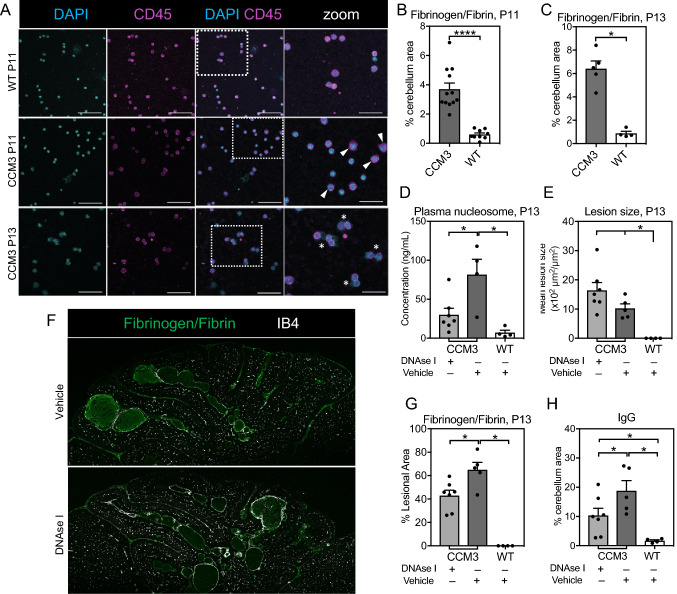
Neutrophil extracellular traps (NETs) and coagulation in the cerebellum and the circulation in *Ccm3*^*iECKO*^ mice (CCM3) and wild-type controls (WT) at an earlier stage of chronic CCM. Roles of NETs in CCM. **A** Representative staining of circulating cells for DAPI and CD45 from WT (top row) and CCM3 (middle and bottom row) mice at P11 by cytospin technique. Triangles indicate activated neutrophils with increased size but have not yet undergone NETosis. Asterisk indicate neutrophils that have released their DNA. Scale bars of DAPI, CD45 and merge panels are 50 μm. Scale bars of zoom panels are 25 μm. **B–C** Percentage of cerebellum area positive for fibrinogen/fibrin at P11 (*n* = 9–12 per group) (**B**), and P13 (*n* = 4–5 per group) (**C**) in chronic CCM. **D–H** Effects of DNase I in vivo treatment in chronic CCM regarding the following: (**D**) Levels of plasma nucleosomes, (**E**) Size of CCM lesions, (**G**) Area of lesions positive for fibrinogen/fibrin, and (**H**) percentage of non-lesional area of cerebellum positive for IgG, at P13. *n* = 4–7 per group. **F** Representative immunofluorescence image showed the staining of fibrinogen/fibrin (green) and isolectin B4 (white) in the cerebellum at P13 for chronic CCM. *P < 0.05; **P < 0.01; ***P < 0.001 for comparisons between groups (Mann–Whitney *U* tests)

Having shown elevated levels of neutrophils and NETs in the circulation and in the cerebellum, often in fibrinogen/fibrin containing cavernomas, at P15 in chronic CCM, we next studied NETs and coagulation at an earlier stage of CCM, at P11 and P13. Contrary to the detection of NETs in both the cerebellum (Fig. 7) and the circulation of Ccm3^*iECKO*^ mice at P15 (Fig. 6J), NETs were absent in the cavernomas of *Ccm3*^*iECKO*^ mice at P11 (data not shown). Interestingly, when we analysed peripheral blood from *Ccm3*^*iECKO*^ mice at P11 by cytospin, we observed some activated neutrophils (based on their increased size) that displayed a phenotype similar to neutrophils about to form NETs (as indicated in Fig. 8A). Despite the absence of NETs at P11, we detected fibrinogen/fibrin in cerebellum from *Ccm3*^*iECKO*^ mice already at P11 (Fig. 8B).

At P13, we detected the presence of NETs in the cavernomas of *Ccm3*^*iECKO*^ mice (data not shown) and significantly increased fibrinogen/fibrin levels (Fig. 8C). Consistently, we also detected elevated level of plasma nucleosomes (indicative of NET formation [47–49]) in *Ccm3*^*iECKO*^ mice at P13 (Fig. 8D). NET formation at P13 was visualised by immunostaining of peripheral blood cells, which showed neutrophils with extracellular DNA tails, a characteristic feature of NET formation (Fig. 8A, asterisks).

### *CCM disease progression and fibrinogen/fibrin formation upon DNase I treatment*

To evaluate the contribution of neutrophils to CCM disease development, we performed an *in vivo* depletion of neutrophils during chronic CCM development. At both P13 and P18, we did not detect any effect of neutrophil depletion, neither on the number nor the size of CCM lesions (Supplemental Fig. 12A–D). We also evaluated the role of NETs in CCM by administration of DNase I to degrade NETs *in vivo* during chronic CCM. We showed that DNase I treatment resulted in approximately a 63% reduction in the level of plasma nucleosomes (Fig. 8D). The DNase I treated mice developed CCM with size and number  of CCM lesions comparable to vehicle-treated mice (Fig. 8E, Supplemental Fig. 12E). However, when we stained these mice for fibrinogen/fibrin, we interestingly found that the DNase I treatment reduced the fibrinogen/fibrin coverage of CCM lesions from 65 to 43% (Fig. 8F–G). Furthermore, we detected approximately a 40% reduction in the non-lesional area of cerebellum positive for IgG (Fig. 8H), indicating that the DNase I treatment reduced vascular permeability.

### *Detection of NETs in the cavernomas of patients with CCM*

**Fig. 9:**
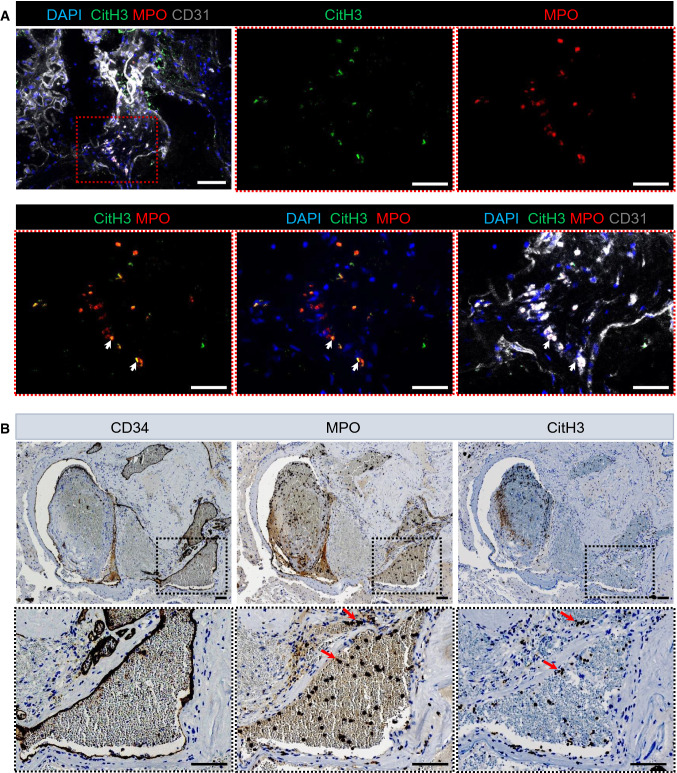
Deposition of neutrophil extracellular traps (NETs) in cavernoma in patients with CCM. **A** Representative immunofluorescence for citrullinated histone H3 (green), MPO (red), DAPI (blue) and CD31 (grey). **B** Representative haematoxylin and eosin staining of brain sections for CD34, MPO and citrullinated histone H3. Scale bars: 100 μm **A**–**B**

To evaluate the potential clinical relevance of NETs in CCM, we performed both immunofluorescence and immunohistochemistry of NETs in brain biopsies from a healthy control and from patients with CCM. Figure 9A shows immunofluorescence images of a representative patient with sporadic CCM. Clusters of NETs (i.e., DAPI^+^MPO^+^CitH3^+^ ^+^) can be seen for sporadic CCM in the proximity of the endothelium of cavernoma, but not for the healthy control (Supplemental Fig. 13A), similar to what was seen for the *Ccm3*^*iECKO*^ mice (Fig. 7A, B). Figure 9B shows immunohistochemistry images of a representative patient with familial CCM. There were also  clusters of NETs in the immunohistochemical analysis of the brain biopsies for familial CCM (Fig. 9B), but not for the healthy control (Supplemental Fig. 13B).

## Discussion

This investigation describes various inflammatory changes that take place during CCM development, including chemokines and cytokines released within the brain tissue, which resulted in immune cell recruitment, and the subsequent local ‘NETosis’. The transcriptomic profiles of endothelial cells from mice with endothelial-specific deletion of the *Ccm3* gene were analysed for both acute and chronic CCM, which showed up-regulation of CCM-associated immune genes that encode proinflammatory cytokines and chemokines, as well as endothelial adhesion molecules. Multiplexed, high-sensitivity, quantitative cytokine assays confirmed that there were increased levels of these cytokines and chemokines in both acute and chronic CCM, and that their levels correlated with lesion size as an indication of disease severity. Scanning electron microscopy, immunofluorescence and flow cytometry analysis further showed recruitment of different leukocyte subsets to CCM lesions. Among these, neutrophils showed the most prominent increases in cell numbers, which occurred already at the early phase of disease progression in the chronic model (P11). We further showed higher frequencies of activated neutrophils and neutrophil–platelet aggregates in the blood in CCM, which might be associated with the higher levels of plasma NETs in CCM, and their deposition in cavernoma. Finally, we demonstrated the clinical relevance of our findings, through confirmation of the presence of NETs in cavernomas of patients with CCM.

Similar to previous studies in *Ccm3*^*iECKO*^ mice [22, 23], we detected many CCM-associated immune genes that encode proteins with various inflammation and immune-related roles in brain microvascular endothelial cells. Some of the roles identified in this study include leukocyte recruitment, mediating the interactions of endothelial cells with leukocytes and platelets, and acting as proinflammatory cytokines. We further showed through previously published scRNA-seq data that venous/venous capillary endothelial cells were the most inflammatory/immune responsive under CCM condition compared with all other endothelial cell subtypes. Since it has been indicated by the scRNA-seq data that endothelial cells of venous and tip cell origin are responsible for cavernoma formation [21], it will be interesting to investigate further regarding if and how the immune genes (*Lbp*, *Cd55* and *Vcam1*) identified in this study that are associated with venous/venous capillary endothelial cells contribute to cavernoma formation. Our group has earlier observed [17] that the expression level of VCAM1 increased in CCM1 lesions. Furthermore, it is noteworthy that the arterial endothelial cells are known for their resistance to lesion formation [21]. Here we show that they are also resistant towards an inflammatory response. It is therefore possible that the ‘protective’ genes expressed in the arterial endothelial cells also play an important role in inflammation.

Using multiplexed, high-sensitivity, quantitative cytokine assays, we showed increased levels of the proteins encoded by many of these CCM-associated immune genes in the cerebellum in CCM. It is encouraging, although not surprising, to see that four of the seven cytokines and chemokines identified in the present study (i.e., IL-1B, IL-6, TNF, CCL2) were shown to reflect CCM disease activities in a study on plasma biomarkers [35, 36]. Future studies can investigate the potential use of the other three cytokines and chemokines identified in the present study as plasma biomarkers (i.e., CXCL1, CXCL2, CCL3), all of which showed strong correlation with CCM lesion size. What makes these findings even more relevant is that many of these cytokines/chemokines are known mediators of the endothelial TLR4 signalling pathway [50], which has recently been shown to play important roles in CCM formation [34].

Neutrophils are among the first leukocytes that are recruited to inflammatory sites. Neutrophils have various roles, which include phagocytosis and the production of reactive oxygen species, proteases and NETs [43]. Here we identified neutrophils as the immune cell type being recruited to the cerebellum in large numbers at an early stage of CCM development. When we performed blood immunophenotyping in wild-type and *Ccm3*^*iECKO*^ mice, we found that *Ccm3*^*iECKO*^ mice showed higher frequencies of circulating activated neutrophils [43], which are also known as low-density granulocytes [42, 43]. This subset of neutrophils has been described as proinflammatory and shown to generate more NETs under certain pathological conditions [43]. This is consistent with our findings of higher levels of NETs in the circulation and within the brain in these mice with chronic CCM. While one should be cautious in making a direct comparison between circulating neutrophils, neutrophils locally in the brain, and neutrophils in cavernomas, it is conceivable that circulating neutrophils become activated as a result of specific local inflammatory changes in the cerebral cavernoma due to focal localisation of defective lesion endothelial cells in CCM. Detection of NETs in biopsies of patients with sporadic and familial CCM further highlights the involvement of activated neutrophils in patients with spontaneous or genetic development of cavernoma.

The clinical role  of NETs in CCM is to date unknown. Here, we have shown in experimental CCM the correlation between levels of inflammatory cytokines and disease severity as measured by lesion size. The inflammatory role of neutrophils and their capacity to produce NETs and release extracellular DNA upon activation was investigated. We found significantly reduced fibrinogen/fibrin deposit within the vascular lesions after DNase I treatment. Similar to neutrophil depletion, this short-time DNase I treatment had no impact on CCM lesion size or number. Nevertheless, DNase I treatment resulted in a significant reduction in circulating nucleosomes and improved barrier function of the lesion vasculature, as shown by reduced IgG leakage. The DNase I treatment is limited to a few days due to the production of neutralising antibodies against DNase I due to its bovine origin [51]. In principle, DNase transgene CCM mice could possibly circumvent this drawback. The contribution of NETs as a potential biomarker in clinical CCM remains to be evaluated in future explorative studies. In addition, we provide data that suggest that these NETs can be  involved in immuno-thrombosis in CCM, although the exact mechanisms behind this remains unclear.

During infection, NET formation is important to maintain host defence functions through the immobilising and killing of invading microbes, thus defining the process known as NETosis [52]. NETosis can be triggered by a variety of stimuli, which include microorganisms, proinflammatory cytokines, and activated platelets and endothelial cells [53]. Excessive NETosis can, however, be detrimental. If dysregulated, excessive NETosis can result in endothelial damage and tissue injury due to their cytotoxic and prothrombotic capacities [54, 55]. Therefore, it will be of great interest to further investigate the possible pathological roles of activated neutrophils and NETosis in CCM patients by analysing NET levels in patient-derived blood samples and correlate to magnetic resonance imaging. Furthermore, with the knowledge of an inflammatory profile in CCM, pharmacological therapies aiming at dampening of inflammation such as non-steroidal anti-inflammatory drugs would possibly be attractive for this patient group.

In conclusion, we used both transcriptomic and proteomic approaches to show increased expression of inflammation-related genes and proteins with different immune functions in CCM. We further showed the recruitment of different leukocytes to CCM lesions, with immune cell maturation towards an adaptive immunity profile in chronic CCM. Among the different types of leukocytes, neutrophils showed pronounced increases in numbers, accompanied by higher levels of NETs in both blood and brain from mice with CCM and in brain biopsies from CCM patients. Degradation of NETs by DNase I treatment resulted in reduced coverage of lesions by fibrinogen/fibrin and improved vascular barrier. Future efforts will be directed to an understanding of how leukocytes (e.g., neutrophils) and their products (NETs) can have effects on CCM development, along with their potential use as biomarkers of CCM.

## Methods

### Mice

The generation of the mouse strain with endothelial-cell-specific ablation of the *Ccm3* gene (*Ccm3*^*iECKO*^ mice) were described previously [20, 30]. In short, the *Cdh5(PAC)*-Cre-ER^T2^/*Ccm3*^flox/flox^ mice were generated through breeding of *Ccm3*^flox/flox^ mice with exons 4–5 of the *Ccm3* gene flanked by loxP sites (Taconic Artemis GmbH), with *Cdh5(PAC)*-Cre-ER^T2^ mice. This provided the mouse line with endothelial-specific and tamoxifen-inducible loss of function of the *Ccm3* gene, referred to here as the *Ccm3*^*iECKO*^ mice. The *Cdh5*(PAC)-Cre-ERT2 mouse line was kindly provided by R.H. Adams (Department of Tissue Morphogenesis, Faculty of Medicine, Max Planck Institute for Molecular Biomedicine University of Münster, Münster, Germany). The mice were maintained in the barrier facility of the Rudbeck Laboratory (Uppsala University, Sweden) and were screened for specific pathogens according to the guidelines of the Federation of European Animal Laboratory Science Associations. All of the mice were housed in microisolator cages that contained wood shavings and enrichment, and were kept in a climate-controlled environment with 12-h light/12-h dark cycles. They were fed standard rodent chow with free access to water. All of the work involving mice studies were conducted according to the principles expressed in the Swedish National Board for Laboratory Animals and the European Convention for Animal Care.

### Disease induction

Tamoxifen was first dissolved in ethanol and then diluted in corn oil (cat no. C8267; Sigma-Aldrich) to the final concentration of either 2 mg/mL or 0.083 mg/mL. In the ‘acute’ model, a single intragastric injection of tamoxifen was given at P1 (final dose, 60 μg per mouse). In the ‘chronic’ model, a single intragastric injection of tamoxifen was given at P1 (final dose, 2.5–5.0 μg per mouse). Injection of tamoxifen on postnatal day 1 (P1) induced deletion of the *Ccm3* gene in these *Ccm3*^flox/flox^ mice. The extent of the *Ccm3* gene deletion determined the pace of disease onset and progression [20].

In all studies, the *Ccm3*^*iECKO*^ mice were compared with littermate controls (hereafter referred to as wild-type control mice), which were obtained by either injecting *Ccm3*^*iECKO*^ mice stated above with corn oil (cat no. C8267; Sigma-Aldrich), or by injecting *Ccm3*^*iECKO*^ mice that did not express Cre-ER^T2^ recombinase (as determined by genotyping) with tamoxifen.

### Human CCM brain biopsies

Biopsies from four patients with sporadic CCM were collected at the Department of Neurosurgery, Helsinki University Hospital (Sweden) during their routine surgical treatment, where the decision for surgery was based solely on the patient clinical needs. Healthy control brain samples were purchased (cat no. OCTF-007; Creative Bioarray), and were from an individual with no known neurological conditions.

Paraffin-embedded biopsies from four patients with familial CCM were obtained from the Angioma Alliance’s DNA/Tissue Bank, as defined through a Material Transfer Agreement. Healthy control brain samples were provided by the Research, Development and Education Department at Uppsala University Hospital (Sweden), and were from an individual with no known neurological conditions.

### Protein extraction and multiplexed, high-sensitivity, quantitative cytokine assays

The cerebellum tissue was frozen at − 80 °C as soon as it was collected. For protein extraction, the tissue was transferred into tubes (Precellys CK14 lysing kits) for soft tissue homogenisation, and immersed in 500 µL cold TBS buffer containing: 1% Triton, and Halt Protease and Phosphatase Inhibitor Cocktail, EDTA-free (78,441, Thermo Fisher Scientific). The tissues were homogenised (Precellys Evolution tissue homogeniser) at 4500 rpm, for 2 × 5 s. This is followed by centrifugation for 10 s to remove foam and bubbles. The cerebellum homogenate was then transferred to a new tube and centrifuged at 16,000×*g* for 1 h at 4 °C, after which the supernatant was removed, aliquoted and stored at − 80 °C until further analysis. Protein concentrations were determined using BCA Protein Assay kits (23,225; Pierce Thermo Fisher Scientific), according to manufacturer instructions. The concentrations of the different cytokines in the cerebellum homogenate were determined using multiplexed, high-sensitivity, quantitative assays (V-Plex Mouse Cytokine 19-Plex kits; and Meso QuickPlex SQ 120 kits; Meso Scale Discovery), according to manufacturer instructions.

### Antibodies

For immunofluorescence analysis of the mouse and human materials embedded in the optimal cutting temperature formulation, the following antibodies and reagents were used. Antibodies: goat anti-mouse CD45 (AF114), rat anti-mouse F4/80 (BM8) and goat anti-human/mouse myeloperoxidase (AF3667) (R&D Systems); rabbit anti-human/mouse/rat histone H3 (citrulline R2 + R8 + R17; ab5103) and rat anti-mouse Ly-6B.2 (7/4) (Abcam); rat anti-mouse I-A/I-E (2G9), rat anti-mouse Ly6G (1A8) and rat anti-mouse B220/CD45R (RA3-6B2) (BD Biosciences); goat anti-mouse fibrinogen/fibrin (GAM/Fbg/7S; Nordic Mubio). Biotinylated *Griffonia simplicifolia* lectin I (GSL I) isolectin B4 (B-1205; Vector Laboratories). For immunofluorescence, the secondary antibodies were produced in either donkey or goat and targeted against the appropriate species, with conjugation with AlexaFluor 488, 568 or 647 (ThermoFisher Scientific). Streptavidin, Alexa Fluor 647 conjugate (ThermoFisher Scientific) was used to detect isolectin B4.

For immunohistochemistry analysis of the paraffin embedded biopsies from the Angioma Alliance DNA/Tissue Bank, the following antibodies were used: mouse anti-human CD34 (#IR632; Dako); rabbit anti-human myeloperoxidase (#IR511; Dako); and rabbit anti-human/mouse/rat histone H3 (citrulline R2 + R8 + R17; ab5103; Abcam).

For flow cytometry, the following fluorochrome-conjugated antibodies and reagents were used. Antibodies: anti-CD45 (30-F11), anti-CD19 (6D5), anti-CD11b (M1/70), anti-Ly6c (HK1.4) and anti-ICAM-1 (3E2) (BioLegend); and anti-CD3ε (17A2), anti-CD4 (RM4-5), anti-γδ T-cell receptor (GL3), anti-Ly6g (1A8), anti-CD11c (N418) and anti-NK1.1 (PK136) (BD Biosciences). LIVE/DEAD Fixable Aqua Dead Cell Stain kits were from ThermoFisher Scientific.

### *In vivo *neutrophil depletion

In the first experiment with mice that were sacrificed at P13, after tamoxifen injection at P1 as described above, each mouse was administered intraperitoneally with either 100ug anti-Ly6G (BioCell #BP0075-1) or vehicle (1X PBS; Gibco) starting from P4 or P5, and then every second day from P6 onwards. Treatment groups were assigned randomly. The mice were sacrificed on day P13 and used for analysis.

In the second experiment with mice that were sacrificed at P18-P20, after tamoxifen injection at P1 as described above, each mouse was administered intraperitoneally with either 100 μg anti-Ly6G (BioCell #BP0075-1) or vehicle (1X PBS; Gibco) except the first day of injection when only 100 μg was administered. The injection was performed twice every week starting from P3-P5, and then every second day from P6 onwards. Treatment groups were assigned randomly. The mice were sacrificed on day P18-P20 and used for analysis.

### DNAse 1 treatment

After tamoxifen injection at P1 as described above, each mouse was administered once daily at P10-P12 with either 10U DNAse 1 (Thermo Fisher #EN0521) diluted in 0.9% NaCl or vehicle (0.9% NaCl). Treatment groups were assigned randomly. The mice were sacrificed on day P13 and used for analysis.

### Brain sections

Mice were anaesthetised by intraperitoneal injection of Avertin, and perfused with 1% paraformaldehyde (PFA) in phosphate-buffered saline (PBS). The brains were carefully removed from the skull and post-fixed overnight by immersion in 4% PFA at 4 °C. The brains were washed in PBS the following day and cryo-protected in 30% sucrose overnight at 4 °C, and then embedded in optimal cutting temperature formulation (Thermofisher Scientific) and frozen at − 80 °C. Sagittal Sects. (7 μm) were prepared using a cryostat (CryoStar NX50; Thermo Scientific) at − 20 °C, and mounted on positively charged glass slides (Thermo Fisher). For immunostaining with antibodies against Ly6g (1A8) and histone H3 (citrulline R2 + R8 + R17; ab5103), the mice were not perfused with PFA. Once removed from the skull, the brains were immediately snap-frozen at − 80 °C, and cryosections (7 μm) were prepared and mounted as described above.

For in vivo neutrophil depletion experiment, mouse brains were obtained and fixed in 4% PFA overnight at 4 °C as described above. Mouse brains were then subsequently embedded in low temperature melting agarose and sectioned as previously described [56]. Briefly, using vibration microtome (HM 650 V; Thermo Scientific) 100-μm-thick sections were sagittal cut from the mid of the cerebellum (position Bregma 0).

The snap-frozen patient biopsies from Helsinki University Hospital were embedded in optimal cutting temperature formulation (TissueTek Sakura). Cryosections (10 μm) were prepared and mounted as described above. Paraffin embedded biopsies from the Angioma Alliance DNA/Tissue Bank were sectioned and stained according to standard protocols, by the Research, Development and Education Department at Uppsala University Hospital.

### Immunofluorescence and microscopy

Cryosections were washed with PBS for 10 min and incubated in blocking/permeabilisation solution (5% donkey serum, 1% bovine serum albumin, 0.3% Triton X-100, in PBS) at room temperature for 1 h. This was followed by incubation with the primary antibody overnight at 4 °C. After washes with 0.01% Triton X-100 in PBS, stained sections were subsequently incubated with the secondary antibody at room temperature for 2 h. DAPI was used for staining of the cell nuclei. Sections were mounted in Fluoromount-G Mounting Medium (ThermoFisher Scientific).

For *in vivo* neutrophil depletion experiment, sections were incubated overnight with the primary antibodies and washed with 0.01% TritonX100 in PBS as described above for cryosections. The sections were subsequently incubated with the fluorophore-conjugated secondary antibodies for 4 h at room temperature, washed, stained with DAPI, post-fixed with 4% PFA and mounted with Fluoromount-G Mounting Medium (ThermoFisher Scientific).

For immunostaining of Ly6g (1A8) and histone H3 (citrulline R2 + R8 + R17; ab5103), the snap-frozen sections were fixed in ice-cold methanol for 10 min, washed in PBS for 10 min, and incubated in 5% bovine serum albumin (for blocking) at room temperature for 1 h, before incubation with the primary antibody overnight, as described above.

### Image acquisition and analysis

Images of whole mouse brains were acquired using a stereo microscope (Leica). All immunofluorescence images of mouse brain and human brain sections (from Helsinki University Hospital) were acquired using a confocal microscope (SP8; Leica) and were analysed using the Fiji software (open source; https://fiji.sc/). To quantify fibrin/fibrinogen, a fixed threshold was set and the percentage of positive signals normalized to lesion area within the cerebellum was analysed. To quantify IgG leakage within cerebellum parenchyma, a mask of the vessels and the lesions was used to exclude signals within the vessel lumen. A fixed threshold was then set and the percentage of positive IgG signals within the cerebellum was quantified. The immunohistochemistry images of the human brain sections from Angioma Alliance were acquired using AxioScan (Zeiss) and analysed using ZEN (blue edition; Zeiss) and QuPath v0.2.3 (https://qupath.github.io/).

For cytospin analysis, neutrophils were identified based on their multi-lobed nucleus. Activated neutrophils with NET-like structures were identified by the presence of extracellular DNA tails around the neutrophils.

### Immune cell isolation from mouse cerebellum for flow cytometry analysis

The mice were anaesthetised with Avertin and underwent intra-cardiac perfusion with cold PBS. The brain was carefully removed from the skull, and the cerebellum was separated from the forebrain. The cerebellum tissue was then minced and dissociated using a dissociator (gentleMACS; 130-093-235; Miltenyi) and the Multi Tissue Dissociation 1 kits (130-110-201; Miltenyi), according to the manufacturer instructions. The cells were resuspended in 5 mL 25% density gradient medium (Sigma-Aldrich), and then centrifuged at 521×*g* for 20 min at 18 °C, as previously described [57]. After centrifugation, the myelin coat and the supernatant were carefully aspirated and discarded, and the cell pellets were washed with washing buffer to remove the density gradient medium [57]. Enriched immune cells were then washed with 10 mL FACS buffer, and stained for flow cytometric analysis.

### Endothelial cell isolation from mouse cerebellum for RNA-seq analysis

For mouse brains collected at P9 and P30 for RNA-seq analysis of acute and chronic CCM, no perfusion was performed and mice were sacrificed by cerebral dislocation after they were anaesthetised with Avertin. In both acute and chronic CCM, mouse brain was carefully removed from the skull, and the cerebellum was separated from the forebrain. Endothelial cells were isolated from the brains (Adult Brain Dissociation kits; 130-107-677; Miltenyi), following the manufacturer instructions. Briefly, the brain tissue was first mechanically and enzymatically dissociated (gentleMACS Octo dissociator; 130-093-235; Miltenyi Biotech), and then myelin, cell debris and erythrocytes were removed. Endothelial cells were enriched by depletion of CD45-positive cells (CD45 microbeads; #130-052-301; Miltenyi Biotech) and selection of CD31-positive cells (CD31 Microbeads; #130-097-418; Miltenyi Biotech,). The collected brain endothelial cells were then processed for RNA-seq analysis.

### Flow cytometry

Enriched immune cells (as prepared according to the section ‘Immune cell isolation from mouse cerebellum for flow cytometry analysis’) were resuspended in ice-cold FACS buffer (Ca^2+^-free, Mg^2+^-free Dulbecco’s PBS supplemented with 1% foetal calf serum, 10 mM EDTA), and stained with a saturating concentration of mAbs on 96-well v-bottomed polypropylene plates (BD Falcon). Cells were filtered through 70 μm cell strainers before analysis. Both cell staining (Live/Dead Fixable Aqua Dead Cell Stain) and forward scatter *versus* side scatter plots were used to include only non-necrotic cells. A flow cytometer was used for acquisition (CytoFLEX S; Beckman Coulter), and the data were analysed using FlowJo (Tree Star).

### Scanning electron microscopy

The tissue preparation and image acquisition were previously described [56]. Mice were anaesthetised with Avertin (Sigma) and perfused with electron microscopy fixative [2% PFA, 2.5% glutaraldehyde (EMS, USA), 2% sucrose, 2 mmol CaCl_2_, in PBS, pH 7.4]. After perfusion, the mice were kept in a sealed plastic bag at room temperature for 2 h. The cerebellum was excised and post-fixed in electron microscopy fixative for 72 h at 4 °C. After fixing, the samples were washed with PBS and sectioned at 1–2 mm intervals using a mouse brain matrix (BSMAS005-1, -2; Zivic Instruments) under a stereomicroscope (Wild Herrbrugg, Wild MB, Switzerland). The sections were then post-fixed in 1% unbuffered osmium tetroxide for 1 h. After osmication, the sections were washed in the buffer three times, dehydrated through a series of graded ethanol concentrations, and dried in a critical point dryer (Agar E3000; Quorum Technologies Ltd., East Essex, UK) using liquid CO_2_ as the drying agent. The samples were then mounted on stubs, and coated with gold using a sputter device (K550X; Emitech Ltd., Kent, UK). Images were taken with an electron microscope (XL 30 ESEM; Philips, Eindhoven, The Netherlands) operated at 10 kV accelerating voltage. High-magnification images (above  ×30,000) of the samples (coated) were also obtained by scanning electron microscopy (4500 field emission-SEM; Hitachi) at 20 kV, with the images recorded digitally.

### Quantification of neutrophil extracellular traps in plasma

Blood was drawn from mice by cardiac puncture, with citrate buffer used as anticoagulant (38 mM citric acid, 75 mM trisodium citrate, 100 mM dextrose). The cells were removed from the plasma by centrifugation at 2000×*g* for 10 min at 4 °C. The plasma was aliquoted and frozen at − 80 °C until analysis. To quantify NETs in the mouse plasma, capture ELISA was used, which is based on citrullinated histone H3 association with DNA. For the capture, clear flat-bottomed immuno nonsterile 96-well plates (439454; Thermofisher Scientific) were coated with 2 µg/mL anti-histone H3 antibody (citrulline R2 + R8 + R17; ab5103; Abcam), overnight at 4 °C. The plates were washed three times with PBS, and blocked with 3% bovine serum albumin in PBS for 1 h. After further washing of the plates three times with PBS, 20 µL plasma and 80 µL incubation buffer with the peroxidase-labelled anti-DNA monoclonal antibody (Cell Death Detection ELISA^PLUS^) were added to each well and incubated on a shaker at room temperature for 2 h. The plates were washed three times with incubation buffer, and then incubated with 3,3',5,5'-tetramethylbenzidine (T0440; Sigma-Aldrich) in the dark. Stop solution (423001; Biolegend) was added to terminate the signal development. The absorbance was determined at 450 nm, with wavelength correction at 540 nm (Synergy HTX multi-mode reader; Biotek).

### Cytospin analysis

Blood samples were obtained via cardiac puncture with citrate buffer used as anticoagulant (38 mM citric acid, 75 mM trisodium citrate, 100 mM dextrose). Plasma was isolated by centrifugation at 4500×g for 15 min at 4 °C. The pellet was used for cytospin analysis. Following centrifugation, cell pellets were resuspended and incubated in RBC buffer (Miltenyi Biotec #130-094-183) for 10 min at room temperature to lyse the erythrocytes. Wash buffer containing 2% FCS (Promo cell #C-37310) in PBS (Gibco #10010-015) was then added to stop the reaction and the samples transferred to ice. The samples were centrifuged at 400 g for 10 min at 4C. The supernatant was removed and the pellet was resuspended with wash buffer and transferred to microscope slides using the Cytospin 4 Centrifuge (Thermo Scientific). Samples were left at room temperature overnight to dry, fixed with ice cold methanol for 10 min and stored at 4 °C.

### Nucleosome ELISA

Blood samples were obtained via cardiac puncture, and plasma isolated as described above. Nu.Q H3.1 ELISA assay (Volition #H310400086920210713A,) was used in the detection of plasma nucleosome according to manufacturer's instructions.

### RNA isolations, RNA library preparation and sequencing

RNA from freshly isolated endothelial cells (CD31^+^, CD45^−^) was prepared using RNeasy micro kits (Qiagen) with an automated system QIACube (Qiagen). For P9 samples, RNA libraries were prepared for three independent biological replicates. RNA sequencing libraries were prepared with the SMARTer Stranded Total RNA sample prep kits—mammalian kits (Clontech), according to the manufacturer instructions. For P30 samples, RNA libraries were prepared for five independent biological replicates with the stranded RNA library prep kits (Collibri; for Illumina), with rRNA depletion kits used according to the manufacturer instructions (#A39003024, A39003096; Invitrogen). The prepared libraries were then analysed with the high sensitivity DNA kits (Agilent) for validation of fragment size and quality control of the amplified RNA libraries. All of the samples were combined in a pool with an equimolar amount of dsDNA. The pool was run on a single lane (paired-end sequencing, 125 bp reads) and sequenced (HiSeq2500 sequencing system, v4 chemistry; Illumina) at the Swedish National Genomics Infrastructure, Uppsala Node, SNP&SEQ technology platform (Science for Life Laboratory, Uppsala). Each sample received ~ 30 million paired-end reads.

### RNA-seq data analysis

#### Reads trimming and mapping

The quality control and prepossessing of the raw reads were done using.

Trim Galore (http://www.bioinformatics.babraham.ac.uk/projects/trim_galore/). In addition, five nucleotides from both 5’ and 3’ end of the reads were clipped to remove unwanted bias. Then trimmed reads were mapped to the mm10 mouse genome (~ 50% unique mapping rate) using default settings of STAR (version 2.5.3a). The number of reads per gene were also counted while mapping by STAR.

#### Differential gene expression analysis

An R package DESeq2 [58] was used to identify differentially expressed genes (DEGs) between two conditions with a cutoff *p*_*adj*_* < *0.05 and |log_2_foldchange|> 0.5. The gene expression read counts were normalized using size factor based method from R package DESeq2. To visualize different gene expression levels in heatmap, normalized expression values were rlog (regularized logarithm)-transformed, which produces transformed data on log2 scale. Then Z-scores are calculated for each gene using rlog-transformed value and used for plotting in the heatmap to avoid the overwhelming of expression values over patterns. The formula for calculating a z-score is is *z* = (x-μ)/σ, where x is the rlog-transformed value for each gene, μ is the rlog-transformed value mean, and σ is the rlog-transformed value standard deviation.

The red color lines for each biological term is to indicate the locations of this biological term related genes in the heatmap.

#### Annotation of differentially expressed genes

The gene set collections—“Gene Ontology”, “Hallmark” and “KEGG”—were downloaded from the Molecular Signatures Database (MsigDB v5.2) and differentially expressed genes (DEGs) were annotated with associated MSigDb gene set ID in those three collections.

#### Identification of CCM-associated immune genes

CCM-associated immune genes are defined as differentially expressed genes (DEGs) that are associated with the following immune-related MSigDb gene sets:

• GO_INFLAMMATORY_RESPONSE
• GO_LEUKOCYTE_MEDIATED_IMMUNITY
• GO_LEUKOCYTE_CELL_CELL_ADHESION
• GO_LEUKOCYTE_DIFFERENTIATION
• GO_REGULATION_OF_LEUKOCYTE_MIGRATION
• GO_REGULATION_OF_LEUKOCYTE_PROLIFERATION
• GO_REGULATION_OF_LEUKOCYTE_ACTIVATION
• GO_REGULATION_OF_CYTOKINE_PRODUCTION
• GO_REGULATION_OF_ADAPTIVE_IMMUNE_RESPONSE
• GO_POSITIVE_REGULATION_OF_LEUKOCYTE_MIGRATION
• GO_REGULATION_OF_LEUKOCYTE_MIGRATION
• GO_NEGATIVE_REGULATION_OF_CYTOKINE_PRODUCTION
•GO_POSITIVE_REGULATION_OF_LEUKOCYTE_MEDIATED_IMMUNITY

#### Functional analysis

R package clusterProfiler [58] was used for GO over-representation analysis with a cutoff of *p*_*adj*_* < *0.05 and redundant enriched GO removed.

Gene set enrichment analysis was performed by Gene Set Enrichment Analysis (GSEA) software [59, 60] using the gene sets from the Molecular Signatures Database (MsigDB v5.2). GSEA performed 1000 permutations. The maximum and minimum sizes for gene sets were 5 and 1000, respectively. The cutoff for significant gene sets was false discovery rate < 25%.

#### Spatial transcriptomics data analysis

The spatial transcriptomics data were obtained our previous study [56]. In summary, two coronal brain sections from a wild-type P8 mouse and two coronal brain sections from an acute CCM P8 mouse were sequenced using 10 × Visium Spatial platform. The sequencing data and histology images were first processed using the software Space Ranger (v1.0, 10 × Genomics), then the pipeline from R package Seurat (v.3.9) was used to perform integrated analysis.

The spots were colour-coded by cluster and overlaid on tissue staining images to identify the spots/clusters that belong to the cerebellum region. Then spots in the cerebellum region were analyzed for their expression levels of *Icam1*, *Cxcl1* or *Ccl2*.

### Statistical analysis

Statistical analysis was performed using GraphPad Prism 8. Fisher’s exact tests were used to determine statistically significant differences in the immunohistochemical detection of the neutrophil numbers between cavernoma without and with fibrinogen/fibrin signals. For correlation of the levels of cytokines with lesion size, Spearman rank correlation coefficient, ρ, and the corresponding P value were calculated for every dataset.

Quantitative data from the multiplex immunoassay and flow cytometry assay were evaluated for normality by D’Agostino & Pearson normality test and Shapiro–Wilk normality test. However, since some data groups did not pass the normality test (alpha = 0.05) and the number of mice in some groups were too low to perform normality test, non-parametric statistical tests were used in all such comparisons for consistency. Kruskal–Wallis test ANOVA with Dunn’s multiple comparisons test were used to determine statistically significant differences in the levels of cytokines and chemokines between different time points. Statistical analyses between *Ccm3*^*iECKO*^ and WT mice were carried out using Mann–Whitney *U* tests.

## Supplementary Information

Below is the link to the electronic supplementary material.

Supplementary file1 (PDF 4975 KB)

## Data Availability

Upon acceptance for publication all sequencing data and material will be available. A database for browsing the data will be accessible upon reviewers’ request.
