# Correction: Inflammation and neutrophil extracellular traps in cerebral cavernous malformation

**DOI:** 10.1007/s00018-022-04418-8

**Published:** 2022-06-29

**Authors:** Anthony C. Y. Yau, Maria Ascencion Globisch, Favour Chinyere Onyeogaziri, Lei L. Conze, Ross Smith, Suvi Jauhiainen, Monica Corada, Fabrizio Orsenigo, Hua Huang, Melanie Herre, Anna-Karin Olsson, Matteo Malinverno, Veronica Sundell, Behnam Rezai Jahromi, Mika Niemelä, Aki Laakso, Cecilia Garlanda, Alberto Mantovani, Maria Grazia Lampugnani, Elisabetta Dejana, Peetra U. Magnusson

**Affiliations:** 1 Department of Immunology, Genetics and Pathology, The Rudbeck Laboratory, Uppsala University, Dag Hammarskjoldsv. 20, 751 85 Uppsala, Sweden; 2 Vascular Biology Unit, The FIRC Institute of Molecular Oncology Foundation, Milan, Italy; 3 Department of Medical Biochemistry and Microbiology, Uppsala University, Uppsala, Sweden; 4 Department of Neurosurgery, University of Helsinki and Helsinki University Hospital, Helsinki, Finland; 5 Department of Biomedical Sciences, Humanitas University, Milan, Italy; 6 IRCCS Humanitas Research Hospital, Milan, Italy; 7 The William Harvey Research Institute, Queen Mary University of London, London, UK; 8 Mario Negri Institute for Pharmacological Research, 20157 Milan, Italy

## Correction to: Cellular and Molecular Life Sciences (2022) 79:206 10.1007/s00018-022-04224-2

In the published article Fig. 1 contain error. The correction Fig. 1 is as follow.

**Fig. 1:**
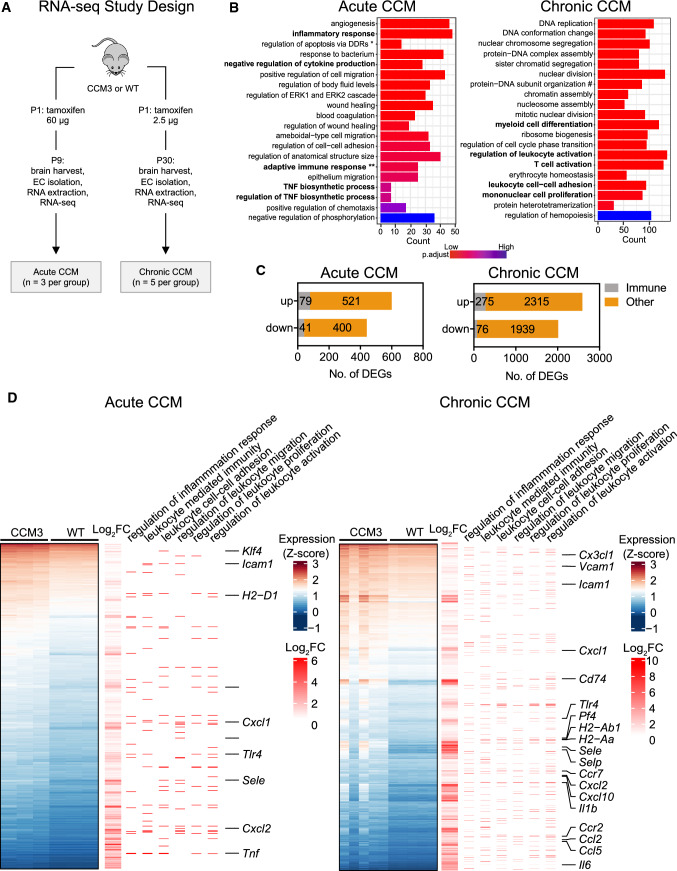
RNA-Seq analysis showed increased expression of inflammation-related genes in brain microvascular endothelial cells from *Ccm3*^*iECKO*^ mice (CCM3) in acute and chronic CCM. **A** Schematic diagram of the experimental design of the transcriptomic study in acute and chronic CCM, comparing CCM3 mice and wild-type (WT) mice (*n* = 3 per group in acute CCM, *n* = 5 in chronic CCM). **B** RNA-Seq gene expression analysis of up-regulated genes showing the top 20 most enriched gene ontology (GO) terms in acute (left panel) and chronic (right panel) CCM. Immune-related GO terms are given in bold. *Regulation of extrinsic apoptotic signaling pathway via death domain receptors, **adaptive immune responses based on somatic recombination of immune receptors built from immunoglobulin superfamily domains, # protein-DNA complex subunit organisation. **C** Bar plots for the numbers of CCM-associated immune genes that were up-regulated or down-regulated (DEGs) in acute (left) and chronic (right) CCM. For definition of immune genes, see Methods. **D** Heatmap showing the expression levels (Z-score of regularized log (rlog)-transformed counts) of significantly up-regulated DEGs (*p*_*adj*_* < *0.05 & |log_2_foldchange|> 0.5; blue: low; red: high) in both acute (upper panel) and chronic (lower panel) models. The first annotation column to the right indicates differential expression in log_2_ fold changes (red: high; white: low). The second to seventh annotation columns indicates the DEGs associated with enriched immune-like GO terms (stated at the top of the figure) from over-representation analysis. Some of the genes labelled after annotation columns were discussed in the text

The original article has been updated.

