# Supplementary material for: Inflammation and neutrophil extracellular traps in cerebral cavernous malformation
Source: Cell Mol Life Sci. 2022 Mar 25;79(4):206. doi: 10.1007/s00018-022-04224-2 (PMC8949649; doi:10.1007/s00018-022-04224-2)
Supplement: Supplementary file 1 — Supplementary file1 (PDF 4975 KB) [file 18_2022_4224_MOESM1_ESM.pdf]

Inflammation and neutrophil extracellular traps in cerebral cavernous malformation

Journal name: **Cellular and Molecular Life Sciences**

Authors: Anthony C. Y. Yau[1], Maria A. Globisch[1], Favour Onyeogaziri[1], Ross Smith[1], Suvi Jauhiainen[1], Monica Corada[2], Fabrizio Orsenigo[2], Hua Huang[1], Melanie Herre[3], Anna-Karin Olsson[3], Matteo Malinverno[2], Veronica Sundell[1], Behnam Rezai Jahromi[3], Mika Niemelä[3], Aki Laakso[3,4], Cecilia Garlanda[5,6], Alberto Mantovani[5,6,7], Maria Grazia Lampugnani[2,8], Elisabetta Dejana[1,2,9], Peetra U. Magnusson[1*]

Affiliations:

1. Department of Immunology, Genetics and Pathology, Uppsala University, Uppsala, Sweden
2. Vascular Biology Unit, The FIRC Institute of Molecular Oncology Foundation, Milan, Italy
3. Department of Medical Biochemistry and Microbiology, Uppsala University, Uppsala, Sweden
4. Department of Neurosurgery, University of Helsinki and Helsinki University Hospital, Helsinki, Finland
5. Department of Neurosurgery, Oulu University Hospital and Division of Clinical Neuroscience, Neurosurgery, University of Oulu, Oulu, Finland
6. Department of Biomedical Sciences, Humanitas University, Milan, Italy
7. IRCCS Humanitas Research Hospital, Milan, Italy
8. The William Harvey Research Institute, Queen Mary University of London, London, UK
9. Mario Negri Institute for Pharmacological Research, Milan, 20157, Italy
10. Department of Oncology and Haemato-Oncology, School of Medicine, University of Milan, Italy

*Corresponding author: Peetra Magnusson, Department of Immunology, Genetics and Pathology, The Rudbeck Laboratory, Dag Hammarskjoldsv. 20, 751 85 Uppsala, Sweden, Peetra.magnusson@igp.uu.se, phone +46 723888418

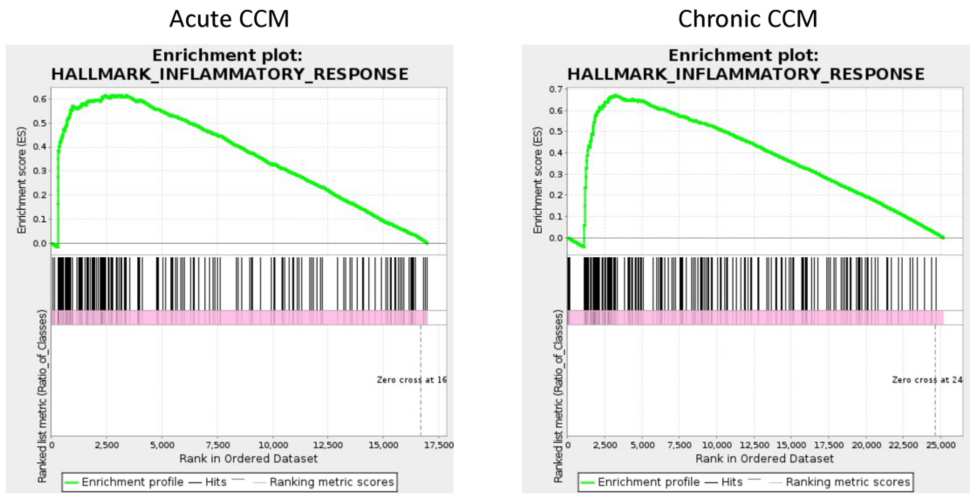

Supplemental Figure 1. **Gene set enrichment analysis showed enrichment of inflammatory response genes in both acute (left) and chronic (right) CCM.**

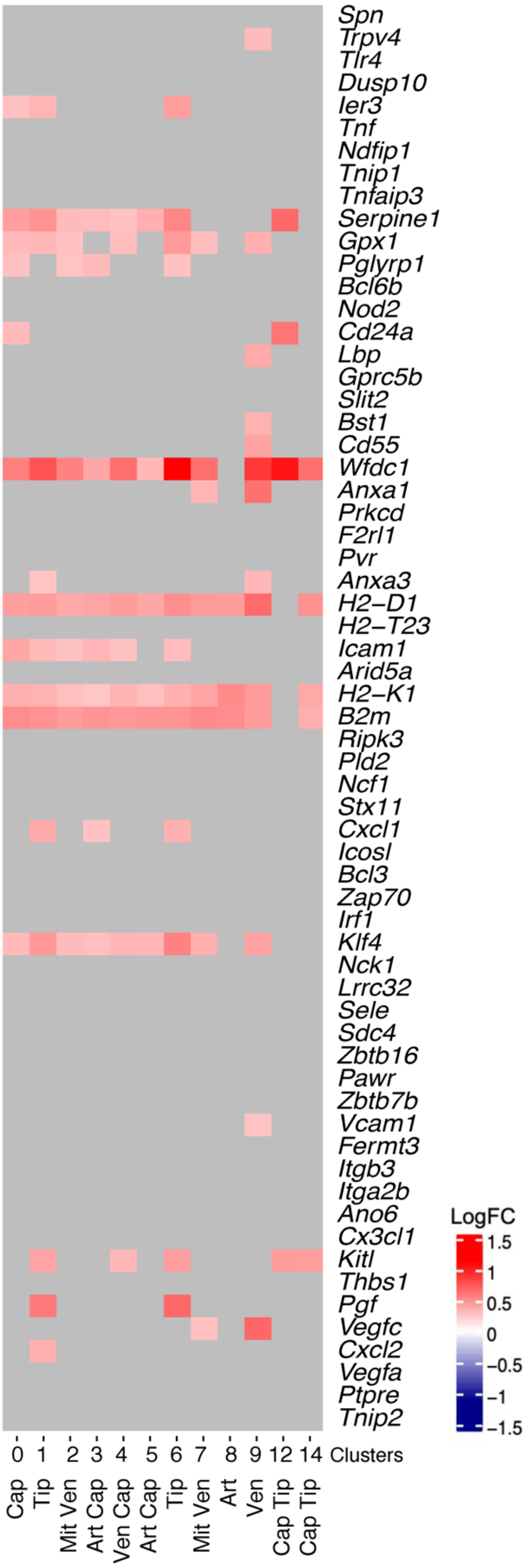

Supplemental Figure 2: Venous/venous capillary endothelial cells showed the highest number of CCM-associated immune genes. Heatmap showing log fold expression changes of all the immune response associated genes identified from bulk RNA-seq (Figure 1D, gene annotation column) in the different brain endothelial cell types of scRNA-seq data. The identity of each endothelial cell subtype cluster (C) is as follows: Cap = capillary (C0), Tip = tip cells (C1, C6), Mit Ven = mitotic/venous capillary (C2, C7), Art Cap = arterial capillary (C3, C5), Ven Cap = venous capillary (C4), Art = arterial (C8), Ven = venous/venous capillary (C9), Cap Tip = capillary/tip cells (C12, C14).

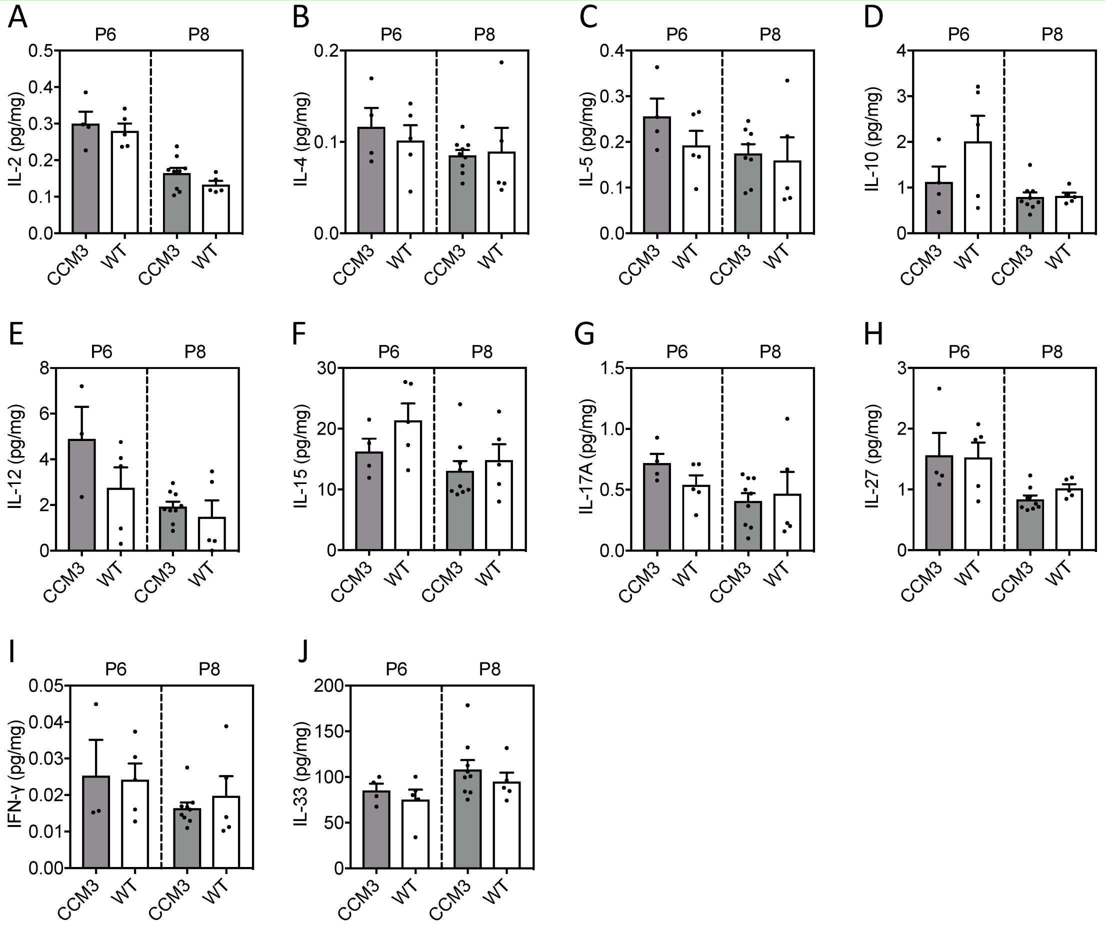

Supplemental Figure 3. Levels of proinflammatory cytokines and chemokines in the cerebellum of wild-type controls (WT) and Ccm3^{iECKO} mice (CCM3) during acute CCM. The following cytokines and chemokines are shown: IL-2 (**A**), IL-4 (**B**), IL-5 (**C**), IL-10 (**D**), IL-12 (**E**), IL-15 (**F**), IL-17A (**G**), IL-27 (**H**), IL-33 (**I**) and IFN-γ (**J**), at P6 (n = 4-5 per group) and P8 (n = 5-9 per group). *, P <0.05; **, P <0.01; ***, P <0.001 for comparisons between groups (Mann–Whitney U tests).

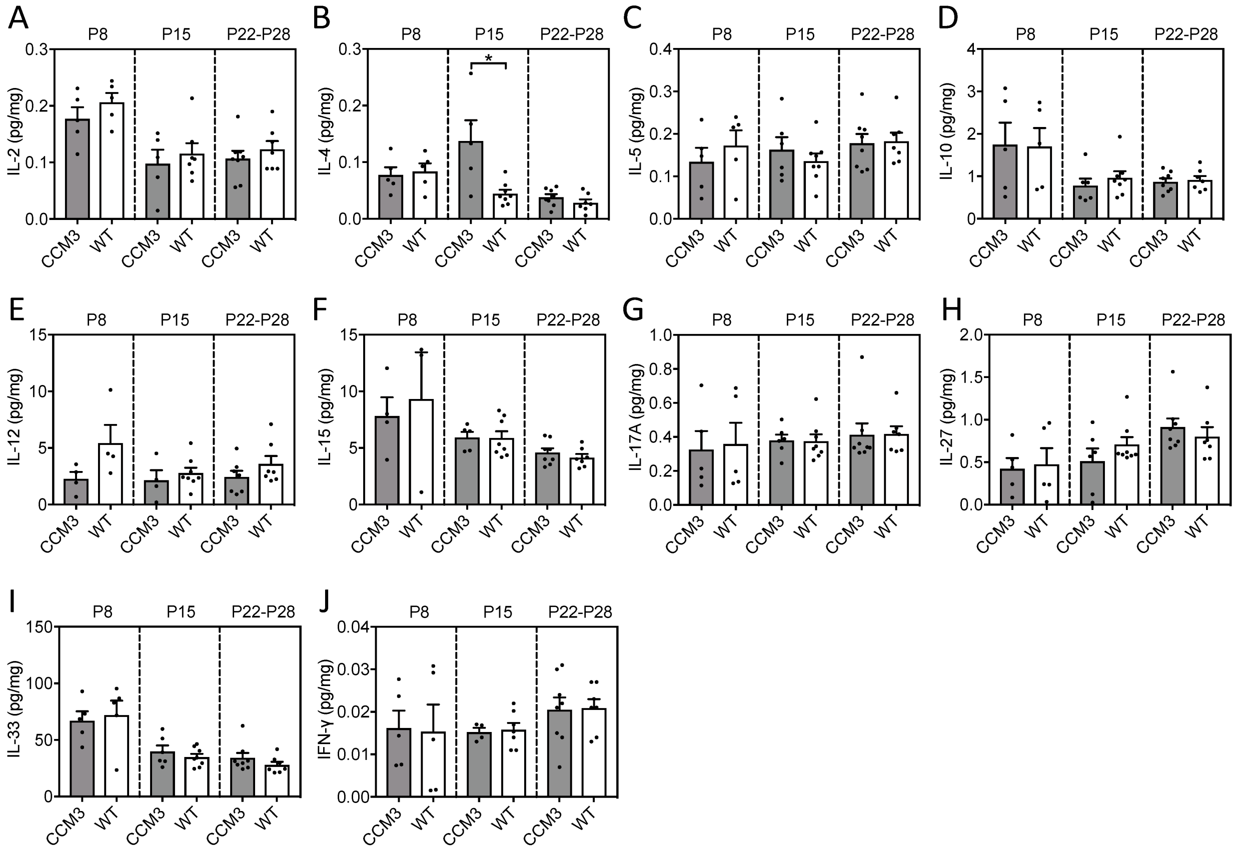

Supplemental Figure 4. **Levels of proinflammatory cytokines and chemokines in the cerebellum of wild-type controls (WT) and Ccm3^{iECKO} mice (CCM3) during chronic CCM**. The following cytokines and chemokines are shown: IL-2 (**A**), IL-4 (**B**), IL-5 (**C**), IL-10 (**D**), IL-12 (**E**), IL-15 (**F**), IL-17A (**G**), IL-27 (**H**), IL-33 (**I**) and IFN-γ (**J**), at P8 (n = 5 per group), P15 (n = 6-8 per group) and P22-P28 (n = 7-8 per group). *, P <0.05; **, P <0.01; ***, P <0.001 for comparisons between groups (Mann–Whitney U tests).

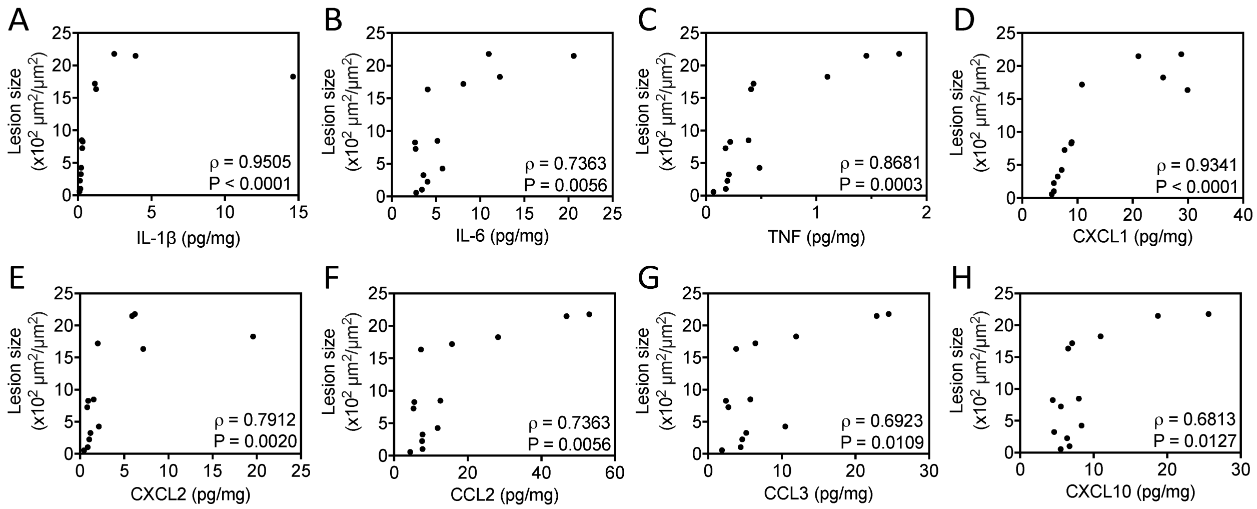

Supplemental Figure 5. Correlations of chronic CCM lesion size with the levels of proinflammatory cytokines and chemokines in the cerebellum of *Ccm3^{iECKO}* mice. The following cytokines and chemokines are shown: IL-1β (**A**), IL-6 (**B**), TNF (**C**), CXCL1/KC/GRO (**D**), CXCL2/MIP-2 (**E**), CCL2/MCP-1 (**F**), CCL3/MIP-1α (**G**) and CXCL10/IP-10 (**H**) (n = 13). To account for any difference in the size of the cerebellum between mice, CCM lesion size is determined by the total area of CCM lesions in the cerebellum in the section (μm^2) / total area of cerebellum in the section (μm^2) x 100. Spearman's correlation coefficient ρ and P values are indicated.

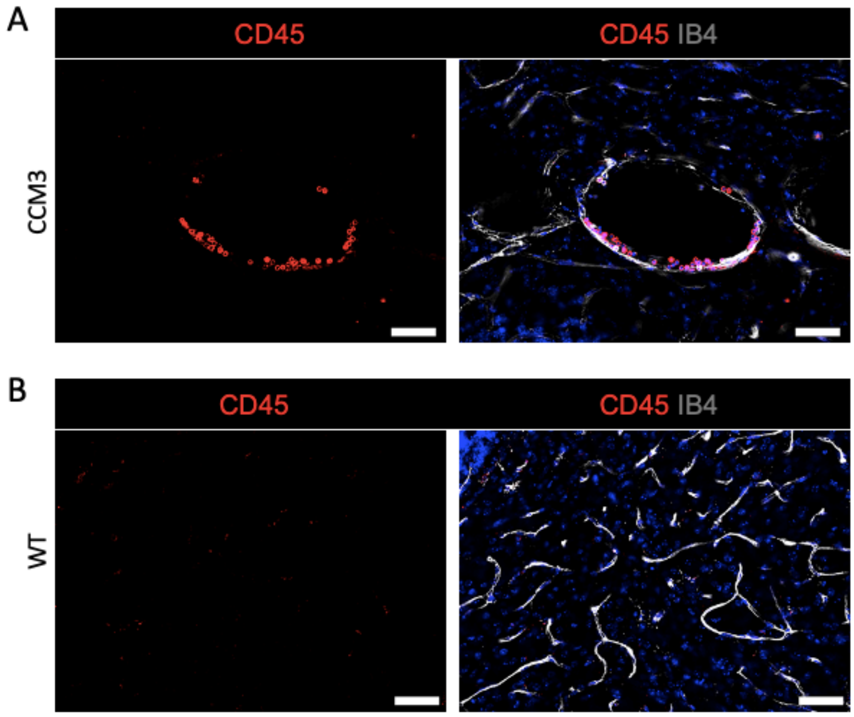

Supplementary Figure 6. Representative immunofluorescence showed recruitment of CD45⁺ leukocytes during chronic CCM. The CD45⁺ leukocytes were only detected at CCM lesions of the *Ccm3*^{iECKO} mice (CCM3) (A), but not in the cerebellum of wild-type controls (WT) (B) at P28 during chronic CCM. DAPI staining (blue), together with CD45 (red) and isolectin-B4 (white). Scale bars: 50 µm.

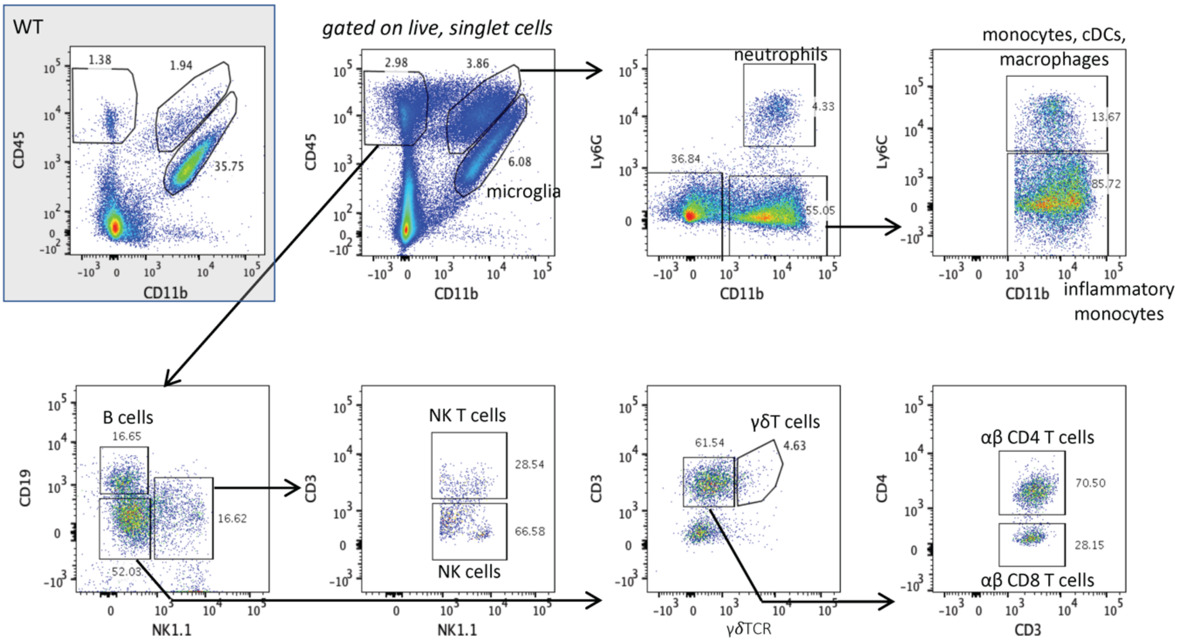

Supplementary Figure 7. **Schematic diagram illustrating the gating of the different immune cell populations by flow cytometry.** These include neutrophils ($CD45^{hi}CD11b^{+}Ly6g^{+}$) , inflammatory monocytes ($CD45^{hi}CD11b^{+}Ly6g^{+}Ly6c^{hi}$), natural killer (NK) cells ($CD45^{hi}CD11b^{-}CD19^{-}NK1.1^{+}CD3^{-}$), CD4 T cells ($CD45^{hi}CD11b^{-}CD19^{-}NK1.1^{-}\gamma\delta TCR^{-}CD3^{+}CD4^{+}$), CD8 T cells ($CD45^{hi}CD11b^{-}CD19^{-}NK1.1^{-}$ $\gamma\delta TCR^{-}CD3^{+}CD4^{-}$) and B cells ($CD45^{hi}CD11b^{-}CD19^{+}NK1.1^{-}$).

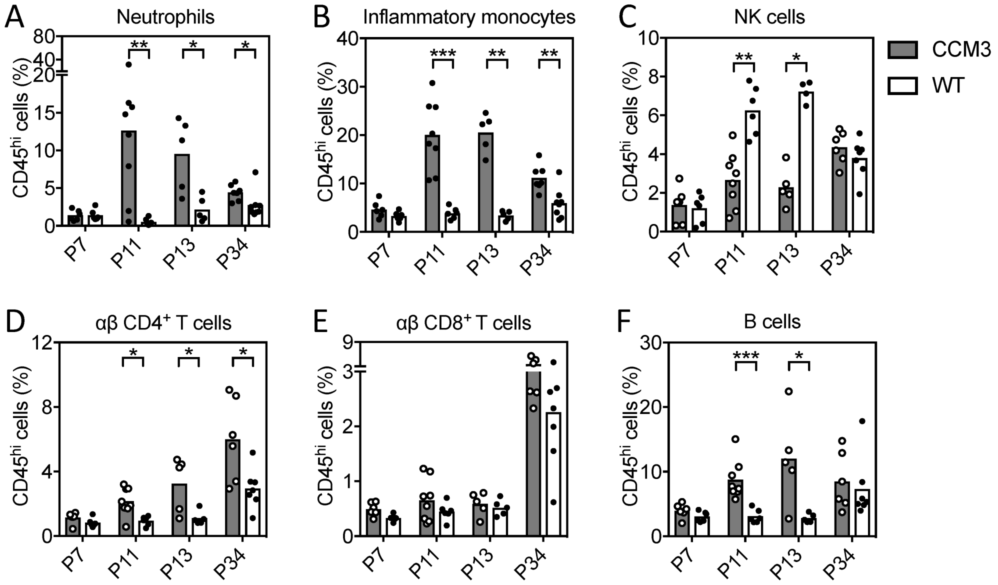

Supplementary Figure 8. **Different immune cell populations as proportions (%) of the total CD45^{hi} immune cells in the cerebellum of the wild-type control (WT) and Ccm3^{iECKO} (CCM3) mice during chronic CCM.** The following immune cell populations are shown: neutrophils (CD45^{hi}CD11b^{+}Ly6g^{+}) (**A**), inflammatory monocytes (CD45^{hi}CD11b^{+}Ly6g^{+}Ly6c^{hi}) (**B**), natural killer (NK) cells (CD45^{hi}CD11b^{-}CD19^{-}NK1.1^{+}CD3^{-}) (**C**), CD4 T cells (CD45^{hi}CD11b^{-}CD19^{-}NK1.1^{-} $\gamma\delta\Delta$TCR^{-}CD3^{+}CD4^{+}) (**D**), CD8 T cells (CD45^{hi}CD11b^{-}CD19^{-}NK1.1$^{-}\gamma\delta$TCR^{-}CD3^{+}CD4^{-}) (**E**) and B cells (CD45^{hi}CD11b^{-}CD19^{+}NK1.1^{-}) (**F**), at P7 (n = 6 per group), P11 (n = 6–8 per group), P13 (n = 5 per group) and P34 (n = 7–8 per group). *, P <0.05; **, P <0.01; ***, P <0.001 for comparisons between groups (Mann–Whitney U tests).

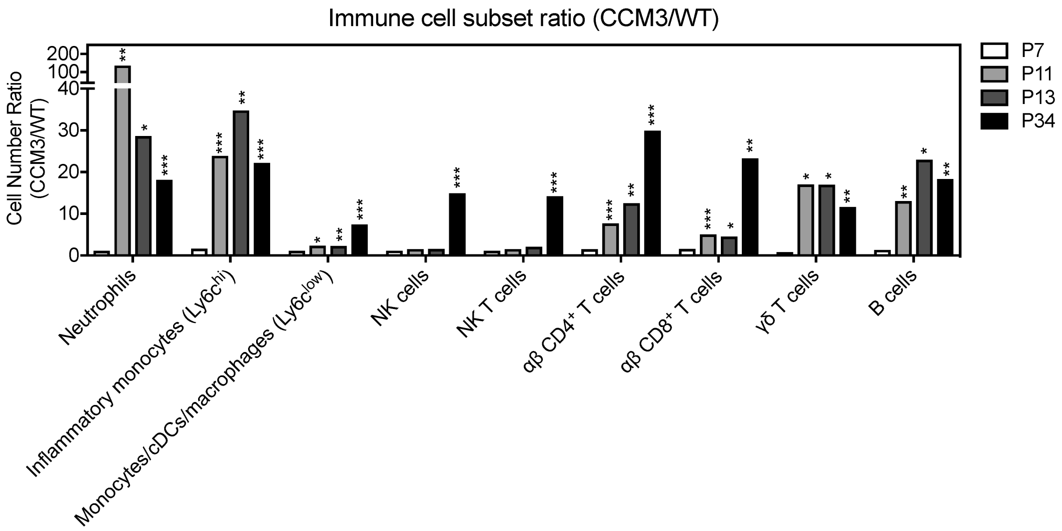

Supplementary Figure 9. **Ratios of different leukocyte subsets *Ccm3[iECKO]*/WT during chronic CCM.** Ratios for number of cells in *Ccm3[iECKO]*/number of cells in WT for the different leukocyte subsets (as indicated) at P7, P11, P13 and P34. *, P <0.05; **, P <0.01; ***, P <0.001 for comparisons between groups (Mann–Whitney U tests).

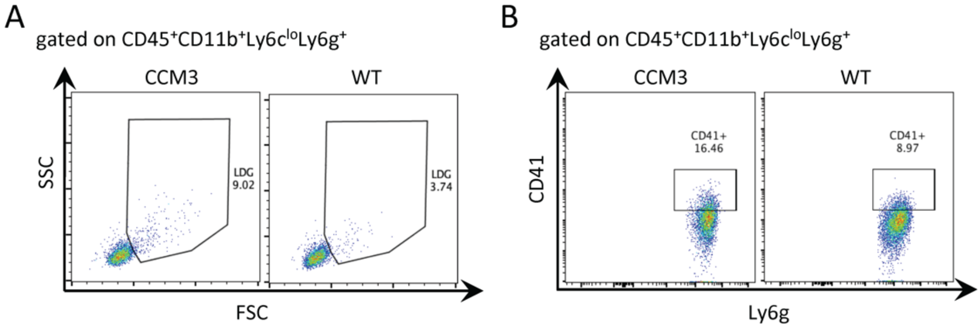

Supplementary Figure 10. Schematic diagram illustrating the gating of activated neutrophils and neutrophil–platelet aggregates in wild-type controls (WT) and *Ccm3^{iECKO}* mice (CCM3) during chronic CCM by flow cytometry. Flow cytometry gating strategy for activated neutrophils/low-density granulocytes (LDG) (**A**) and neutrophil–platelet aggregates (**B**), gated on CD45^{+}CD11b^{+}Ly6c^{lo}Ly6g^{+} cells.

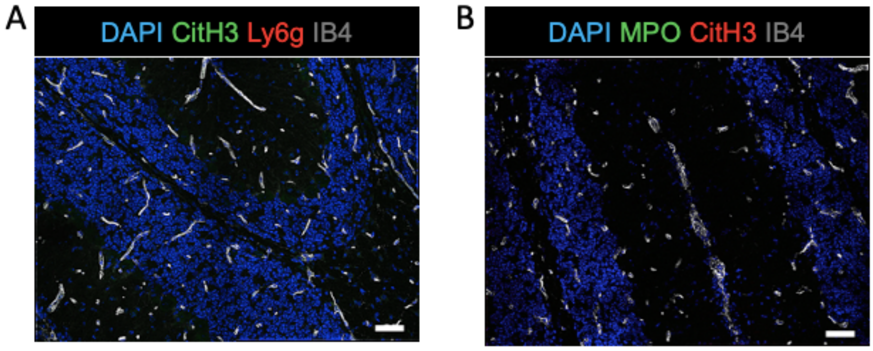

Supplementary Figure 11. **Characterisation of neutrophil extracellular traps (NETs) in mouse cerebellum of wild-type mice.** Representative immunofluorescence showing the absence of NETs in brain sections from wild-type control mouse, at P15. (**A**) Citrullinated histone H3 (green), Ly6g (red), DAPI (blue) and isolectin B4 (grey). (**B**) MPO (green), citrullinated histone H3 (red), DAPI (blue) and isolectin B4 (grey). Scale bars: 50 μm (A-B).

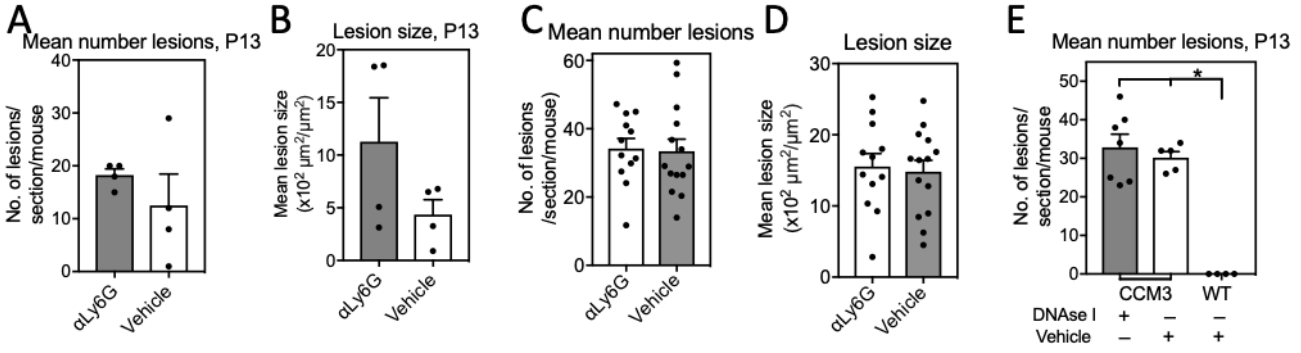

Supplemental Figure 12. The effect of depletion of neutrophils (by anti-Ly6G treatment) and degradation of NETs (by DNase I treatment) on CCM in *Ccm3^{iECKO}* mice as determined by lesion size and number. (A-D) The size (A, C) and number of CCM lesions (B, D) upon treatment of Ly6G determined at P13 (n = 4 per group, A, B) and at P18 (n = 12-14 per group, C, D) in chronic CCM. (E) Effects of DNase I *in vivo* treatment on number of CCM lesions at P13 in chronic CCM. n = 5-7 per group *, P <0.05; **, P <0.01; ***, P <0.001 for comparisons between groups (Mann–Whitney U tests).

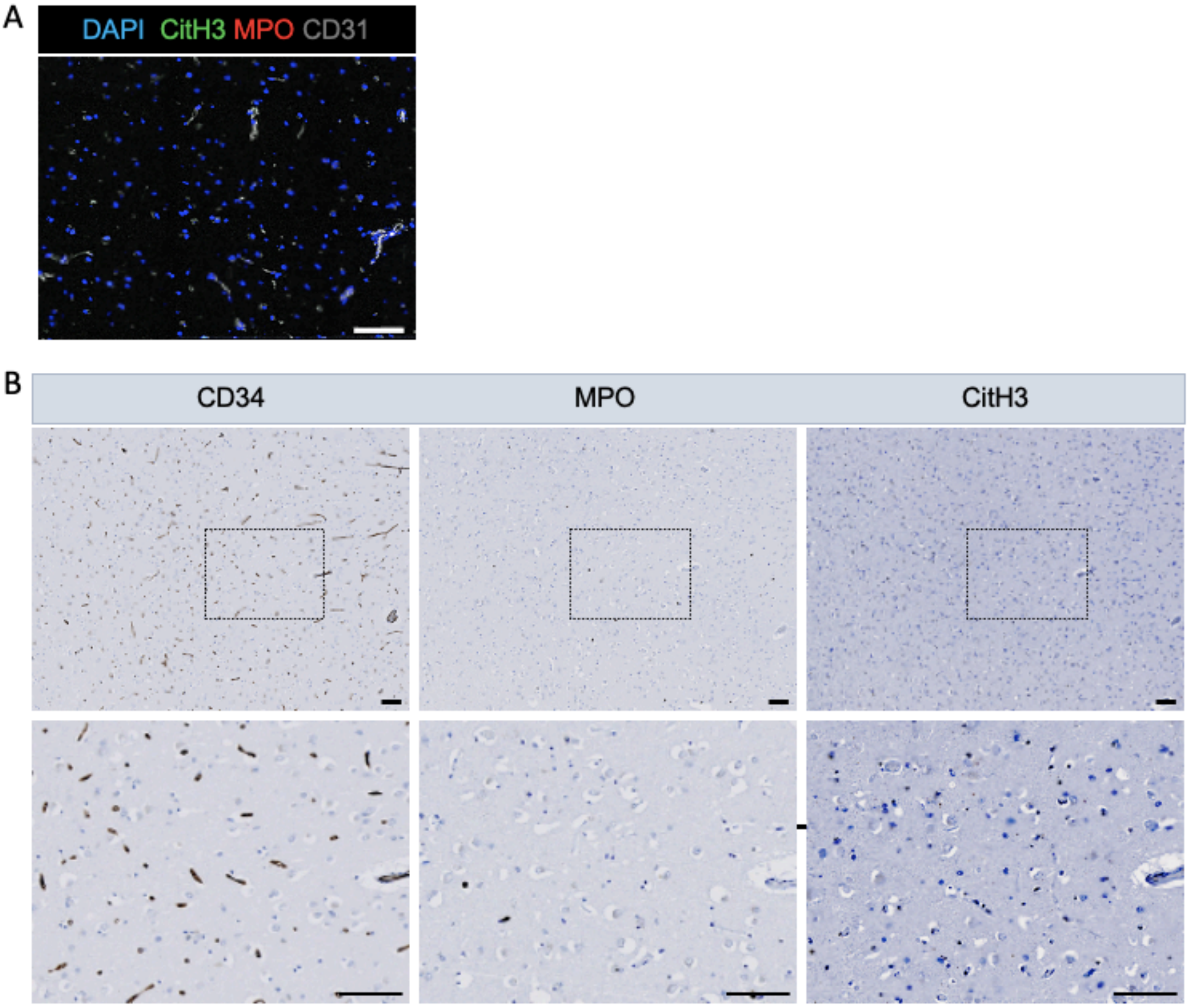

Supplemental Figure 13. Absence of neutrophil extracellular traps (NETs) in brain sections from healthy human. (**A**) Representative immunofluorescence showing the absence of citrullinated histone H3 (green), MPO (red), DAPI (blue) and CD31 (grey). (**B**) Representative haematoxylin and eosin staining of brain sections for CD34, MPO and citrullinated histone H3. Scale bars: 100 µm (A-B).
